# Supplementary material for: Characterising performance information use in the primary healthcare systems of El Salvador, Lebanon and Malawi: multiple qualitative case study protocol
Source: BMJ Open. 2022 Nov 21;12(11):e060503. doi: 10.1136/bmjopen-2021-060503 (PMC9680164; doi:10.1136/bmjopen-2021-060503)
Supplement: Supplementary data [file bmjopen-2021-060503supp001.pdf]

Supplementary file – Interview guidelines

Munar W, *et al. BMJ Open* 2022; 12:e060503. doi: 10.1136/bmjopen-2021-060503

El Salvador interview guidelines- PHC team members

Introduction.

For the past decade, the Salud Mesoamerica Initiative has been working in collaboration with the Ministry of Health to promote maternal and child health in El Salvador. The overall objective of this study is to assess the ways in which MCH performance information is collected and used at the local and national levels in El Salvador. The team obtained non-objection from the Ministry of Health to conduct this research. The findings will be used to develop better health care practices in El Salvador and hopefully contribute to better health outcomes for Salvadorans.

1. Do you recall participating (being interviewed) in 2018 for a study by (anonymized) and the Salud Mesoamerica Initiative?
2. Could you describe to me what you remember about the study (studies) in which you participated?

Section 1 Priorities and goals

1.1 How long have you been in this position?

1.2. What was your previous position?

1.3. Could you describe to me -in broad outline- the main activities you carry out in your current position?

1.4 What do you consider to be the priorities of the Ministry of Health in the primary health care system? What do you think of these priorities?

1.5. What are the priorities of your PHC team at this moment? How do they prioritize activities in the Unit to which you belong?

1.6. What is the role of the PHC teams in the health system of El Salvador?

1.7. Are you familiar with the Mesoamerican Health Initiative Program? If yes, is SMI currently supporting this Unit? Give me an example of how this support occurs? Based on your criteria, what has been the greatest contribution from SMI to this PHC team?

Section 2 - Performance Measurement

2.1. What is your perception of the work your PHC team is currently doing?

2.2. How do you know if your team is doing its job well or not?

2.3 The Salud Mesoamerica Initiative used to measure PHC team performance with some frequency. Rewards and recognition were often given to teams that were positively evaluated. Did you experience these?

Please describe those experiences.

How did you feel about those experiences (performance measurement and recognition)?

Munar W, *et al. BMJ Open* 2022; 12:e060503. doi: 10.1136/bmjopen-2021-060503

2.4. Are there other performance appraisal experiences?

2.5. How is your team's performance evaluated now?

Let's analyze the most recent example.

2.6. When did the appraisal occur?

2.7. Who did the evaluation?

2.8. How often is this done?

2.9. What data sources were used the last time one of these evaluations was conducted?

2.10. In the most recent evaluation of the team's performance, did the team receive any feedback?

2.11. How did you feel about this feedback?

2.12. How do you think the team felt? Elaborate on responses.

If feedback was provided:

2.13. How was the feedback provided? (Oral, written)

2.14. How often is feedback provided to your team about their performance?

2.15. How did you feel about this feedback?

2.16. What was done with the information contained in the feedback?

2.17. Were other team members made aware of the feedback? If yes, how was it shared?

2.18. Did the team do anything to correct their collective performance? Could you give me an example of an activity that was done?

If NO, comments or feedback were provided:

2.19. Would you like feedback on your team's performance?

Why?

2.20. Do you have any meetings with the MOH that you attend to discuss your team's performance?

If YES:

2.21. What is your opinion of these meetings, and why?

2.22. Are comparisons made between your team's performance and the performance of other teams?

Yes/no, please inquire:

2.23. How important would it be for you to attend these meetings? Do you feel it would be important to attend as a team? Why?

Section 3 - First Level of Care team performance management and individual work motivation

3.1. What aspects of your work as part of a PHC team are most rewarding to you?

3.1.1. Tell me about a recent work event that made you feel deeply rewarded or gratified.

3.1.2. Why did you feel this way? (Explore the 'why' in terms of emotions and feelings)

3.2. Sometimes the work is hard. Apart from COVID, what aspects of your work in a PHC team have been negative?

3.2.1. Tell me about a recent work event that affected you negatively.

3.2.2. Why did you feel this way? (Explore the 'why' in terms of emotions and feelings)

Munar W, *et al. BMJ Open* 2022; 12:e060503. doi: 10.1136/bmjopen-2021-060503

El Salvador decisionmakers at national and subnational levels

Introduction.

For the past decade, the Salud Mesoamerica Initiative has been working in collaboration with the Ministry of Health to promote maternal and child health in El Salvador. The overall objective of this study is to assess the ways in which MCH performance information is collected and used at the local and national levels in El Salvador. The team obtained non-objection from the Ministry of Health to conduct this research. The findings will be used to develop better health care practices in El Salvador and hopefully contribute to better health outcomes for Salvadorans.

Section 1 - Current Ministry of Health Primary Health Care Priorities

1.1. Tell me about the Ministry's priorities in the area of primary health care.

1.2. What are the strategic principles that guide the Ministry's PHC priorities?

Probe for:

1.2.1. Urban vs. rural; Comprehensive versus selective.

1.2.3. Efficiency and equity in the allocation.

1.2.4. Universalization versus targeting.

1.3. What role do the PHC teams now have in the functioning of the PHC system?

1.4. How well do you think PHC teams are doing their work?

1.5. What is the current role of SMI in relation to the priorities of the Ministry of Health in PHC?

Probe:

1.5.1. What is your perception of the value of MCH assistance to the MOH?

1.5.2. Could you give me an example?

Section 2 - Measuring PHC Team Performance

2.1 How does the Ministry evaluate the performance of the country's PHC system?

2.2 And in particular, how does the Ministry monitor the activities of the PHC teams?

2.3. Does the Ministry discuss any specific service delivery objectives with the teams?

2.3.1. What role do the teams play in the establishment of these objectives?

2.3.2. Describe the process (Investigate frequency, types of objectives, e.g., scope and/or facilities).

2.3.3. What performance indicators are now used?

Probe for:

- number of indicators used and request a list of key performance indicators in use.

2.3.4. Are performance targets used in all teams in the country? (If only the 75 teams in SMI's area of influence, ask why.)

2.4. In terms of service delivery, how does the Ministry evaluate the performance of the PHC teams?

Probe:

2.4.1. Using a recent example from a specific rural region, describe the process in detail.

2.4.2. What data sources did you use?

2.4.3. Who collects performance data from PHC teams?

2.4.4. Who analyzes the PHC team performance data?

2.4.5. How are the results presented to the PHC teams?

Section 3- Performance Management of PHC Teams

3.1. What are the PHC teams supposed to do with the results of the performance evaluation?

3.1.1. Does the ministry support the teams in understanding the results? How?

3.1.2. The Ministry has a long tradition of convening team managers and regional officers in frequent weekly or biweekly meetings to monitor progress. Is it still done? What are the results?

Section 4: Managing the overall performance of the PHC system

4.1 Beyond performance management of PHC teams, what else is the ministry doing with the results?

Probe:

4.1.1. Are the results used for planning? (If yes, ask for a recent example) 4.1.2.

4.1.2. Are the results used for budgeting? (If yes, ask for a recent example) 4.1.3.

4.1.3. Are the results used for any internal Ministry of Health reporting? (If yes, please ask for a recent example) 4.1.4.

4.1.4. Are the results used for MCH reporting? (If yes, please ask for a recent example) 4.2.

4.2. Does the MOH use aggregated data from PHC teams to adjust its priorities? How? Could you give me examples?

Lebanon interview guidelines - Health service providers and data collectors in the First Level of Care

Section 1- PHC Performance Measures

How is the aggregate performance of PHC system determined? Is there a performance assessment? What does it include?

Can you reflect on the processes and tools through which PHC system performance information is collected and processed? How would you characterize these?

Are there established performance indicators for PHC? If yes, can you share a sample of the performance indicators template (without any patient information or any other personally identifiable data)

How are the performance indicators selected? Which entity/department/unit/platform/team has input in this process (please provide only titles, not individual names)? Are these selected according to explicit criteria including usefulness, scientific soundness, reliability, representativeness, feasibility, accessibility?

Is there a guide/manual on how to calculate and interpret performance indicators? Does reporting on the performance indicators occurs on a regular basis?

How is information about performance measures communicated to you? For example, how did you first hear about them?

When a decision is made at the national level, how is it translated /monitored/implemented at subnational levels? Which entity/department/unit/platform/team is responsible for the communication from national to subnational level (please provide only titles, not individual names)? From subnational back to national level? How quickly does this happen?

What interventions or programs, if any, are implemented to ensure an enabling system environment for quality at level of PHC?

Registration and licensing; External evaluation/accreditation; Clinical governance; Public reporting and comparative benchmarking

Section 2- Performance Targets and Goals

2.1. How are performance targets for PHC set? Which entity/department/unit/platform/team is involved in the process (please provide only titles/department/unit, not individual names)? Are these typically set at a national, sub-national, or sub-regional level or left to the discretion of the facility?

Section 3- Data collection, analysis and sharing

3.1. Data collection

3.1.1. Is the data needed to construct the performance measure feasible to collect?

3.1.2. In what format is data collected? Is there a designated person to enter data/compile reports from the different units in the health facility?

3.1.3. What is the frequency of data collection? Is there a set data submission schedule?

3.1.4. Is there coordination of data collection across all PHC, e.g. via a unique identifier or a centralized data storage platform?

3.2. Data storage

3.2.1. In what database or file do you enter or store the data after you receive them? What computer program do you use? How frequently do you enter/store the data?

3.2.2. Any challenges in entering/storing the data? Any privacy issues when storing data?

3.3. Data aggregation

3.3.1 Do you perform any data aggregations? Which ones? Using which calculations? How often? Are there any challenges in aggregating data?

3.4. Data quality

3.4.1. What quality checks do you perform on the data? Is there a designated person to review the quality of compiled data prior to submission to the next level, e.g., to districts, to regional offices, to the central HMIS, etc.?

3.4.2. Are there any known issues with data quality?

3.5. Data reporting & sharing

3.5.1. Are there written guidelines available on reporting protocols, including the following: what they are supposed to report on; how reports are to be submitted, e.g., in what specific format; to whom the reports should be submitted and when the reports are due?

3.5.2. In what format do you share the data (type of form, file, database, aggregation levels)? Are data visuals prepared (graphs, tables, maps, etc., balanced scorecards) showing achievements toward targets (indicators, geographic and/or temporal trends, and situation data)?

3.5.3. With whom do you share the data? How often do you share the data?

3.5.4. How do you share the data (e.g., internet, email, USB flash drive, disk, hard copy)?

3.5.5. Are there any delays or challenges in preparing data & sending them?

Section 4- Performance Monitoring & Feedback

How is progress towards the performance targets monitored/tracked? Can you give us an example? Is this considered routine?

Is there a performance monitoring or management team?

Which entity/department/unit/platform/team is the most responsible for managing or tracking progress for performance measures? (please provide only titles, not individual names)

Which entity/department/unit/platform/team do they report to/who manages this process? (Please provide only titles, not individual names)

Which entity/department/unit/platform/team is in charge of the data pulls and reports? (please provide only titles, not individual names)

Which entity/department/unit/platform/team do the data analysts and the performance manager report to? (Please provide only titles, not individual names)

Section 5- HIS and data quality assessment

5.1. What specific information flows has the organization in place to support the performance management systems? Are systems interoperable so that information can be shared across geographic areas, types of facilities, etc.?

5.2. Are staff trained in how to use the information systems (not only collecting data, but also collating, analysing and interpreting data)

5.2.1. Do these staff typically have dedicated time allocated for using information systems and interpreting data?

5.2.2. Do they have capacity to explain the implications of the results of data analysis?

5.2.3. Are the information systems accessible to these individuals?

5.3. Are there data quality assessment mechanisms in place?

5.3.1. Are there written instructions/guidelines on how to perform a data quality review or data quality check?

5.3.2. Do data management staff conduct regular checks of the accuracy and completeness of data prior to submitting reports to the next level (using automated electronic checks, where appropriate)?

5.3.3. Does the health facility receive periodic feedback from higher levels (e.g., MOPH) on data quality?

5.3.4. What is your perception of the quality of existing data on PHC performance? How can it be improved?

Section 6- Supervision & Feedback on performance

Do you receive technical support or supervision in your work?

Which entity/department/unit/platform/team conducts supervision? What sort of support of qualifications do these individuals have? (please provide only titles, not individual names)

Which entity/department/unit/platform/team receives supervision? Which ones do not? (please provide only titles, not individual names)

Where do supervisory visits take place and how long do supervisory visits last?

What sort of activities do supervisory visits cover? Does the supervisor use a standard checklist for assessment?

How frequently does supervision occur?

Is there an established schedule for supervision, or do visits need to be requested?

Does the supervisor send report/ written feedback on the past supervisory visit(s)?

Do you get feedback on your performance? How? Frequency? What does it look like? How would you improve this process?

Feedback on data quality (including data accuracy, reporting timeliness, and/or report completeness)

Feedback on service performance based on reported data (e.g., appreciation/acknowledgement of good performance; resource allocation/mobilization)

What, if any, feedback loops exist between the facility level, regional, sub-national level and national level and how do they function?

What rewards — financial and/or non-financial — will managers and other employees gain by achieving performance targets or other assessed aspects of performance (and what penalties will they suffer by failing to achieve them)?'

Section 7- Implementation of quality improvement activities

Are discussions often held to review key performance targets (tracking progress against targets) based on performance measure information?

Are quality improvement activities implemented to take corrective action?

How are the objectives of quality improvement activities set?

Which entity/department/unit/platform/team initiates these activities? (please provide only titles, not individual names)

Which entity/department/unit/platform/team monitors them? (please provide only titles, not individual names)

Section 8- Overall perceptions on collection of performance measure information

8.1. To what extent to do you agree with the following:

I can use data for identifying performance gaps and setting targets

I feel discouraged when the data that I collect/record are not used for taking action (either for monitoring or decision making)

I find collecting/recording data to be tedious (i.e., repetitive or duplicative)

I find that the data that I collect burdens my workload, making it difficult for me to complete my other duties

Collecting data is meaningful/useful for me

I feel that the data I collect are important for monitoring the performance of the health services provided at my facility/unit

My work of collecting data is appreciated and valued by decision-makers at national and subnational level

Lebanon decisionmakers at national and subnational levels

Section 1- PHC Performance Measures

How is the aggregate performance of PHC systems measured (organizational and whole-system level)?

How do you track progress toward goals? Can you show us an example?

Which entity/department/unit/platform/team are most responsible for managing or tracking progress for performance measures? (please provide only titles, not individual names)

Which entity/department/unit/platform/team do they report to/who manages this process? (please provide only titles, not individual names)

Which entity/department/unit/platform/team is in charge of the data pulls and reports? (please provide only titles, not individual names)

Which entity/department/unit/platform/team do the data analysts and the performance manager report to? (please provide only titles, not individual names)

What rewards — financial and/or non-financial — will managers and other employees gain by achieving performance targets or other assessed aspects of performance (and what penalties will they suffer by failing to achieve them)?

Section 2- Awareness of data flow at different levels

2.1. Are you aware of how PHC performance measurement information is collected and processed at different levels?

2.2. Can you reflect on data flow from facility level to district/subnational to MoPH? What is your perception of the process?

Section 3- Data format

3.1. In what format do you receive performance measure information?

3.1.1. Are data visuals (e.g. summary tables, graphs, geographic information system, maps, pivot tables, decision support systems, etc.) showing achievements toward targets (indicators, geographic and/or temporal trends, and situation data) prepared and used to display information at MOPH level and within health facilities. They are up to date and clearly understood

3.1.2. Are reports or bulletins (annual, quarterly, etc.) periodically produced based on an analysis of performance measurement information and distributed to key stakeholders? Do they contain discussions and decisions/recommendations based on key performance targets?

3.1.3. Are there challenges with interpreting the information?

3.2.3. What is the frequency of receiving performance measure information? Is there a set schedule for receiving such information? What is your perception of the process?

3.2. How do you receive the performance measure information (e.g., internet, email, USB flash drive, disk, hard copy)? In what database or file is data stored or entered after you receive them?

3.3. What quality checks do you perform on the data? Are there any known issues with data quality?

3.4. How could (or should) health information products look like for policy-makers? and at what level of disaggregation and/or in which quality?

3.5. With whom do you share the data? In what format do you share the data (type of form, file, database, aggregation levels)? How do you share the data (e.g., internet, email, USB flash drive, disk, hard copy)?

Section 4- Feedback on data received

4.1. When a decision is made at the national level, how is it translated /monitored/implemented at subnational levels? Which entity/department/unit/platform/team is responsible for the communication from national to subnational level (please provide only titles, not individual names)? From subnational back to national level? How quickly does this happen?

4.2. Is feedback systematically provided to all sub-reporting units on the quality of their reporting (that is, accuracy, completeness, and timeliness)?

4.3. Is feedback systematically provided to all sub-reporting units on the use of performance measure information for decision-making?

Section 5- Decision-making process

5.1. How are decisions made at your institution? What factors have strong influence on decision-making process:

Personal preference of decision makers

Superiors' directives

Evidence/facts/data (RHIS data)

Funding directives from higher levels

Political considerations

Official health sector strategic objectives

Locally identified health needs of the population

The relative cost of interventions

5.2. Is there a mandate to use data to inform decision-making? What incentive structures are in place to promote the use of data in decision-making? Are there any consequences for NOT using data to inform critical decision-making?

Section 6- Use of data in decision-making process

6.1. Can you reflect on the processes through which decision makers (at national and district levels) make sense of, and use PHC system performance information? What is your perception of the process?

6.2. Do the existing performance measure information respond to policymakers' priorities and needs? Are data producers and users brought together periodically to discuss ways of making routine data more relevant to policy makers and planners and to enhance the understanding of routine health statistical findings?

6.3. How is aggregate PHC system performance information used by decision makers at the national and district level?

6.4. Were any decisions made based on the discussions of the performance measurement information? Such as:

Advocacy for policy, programmatic, or strategic decisions

Formulation of plans

Budget preparation/reallocation

Medicine supply and drug management

Human resource management (training, reallocation, etc.)

Promotion of service quality/improvement, equity

Section 7- Impact

7.1. Would your organizational priorities be different without performance measures? How? How do measures affect your relationship or interactions with staff?

7.2. How have performance measures changed the way you operate?

7.3. How do performance measures affect policy decisions? Budget decisions? Staff decisions? Scheduling? Education/training/orientation?

7.4. To what extent is (staff) compensation linked to performance measures? Can you explain how that works? How does this affect your perception of performance measures?

Munar W, *et al. BMJ Open* 2022; 12:e060503. doi: 10.1136/bmjopen-2021-060503

Section 8- Perceptions on data use culture

8.1. What is your perception of the prevailing culture for data use at your institution?

Probes:

A culture of information use is promoted by policy leaders and decision-makers, and is reflected in the use of facility and community-based data in planning, monitoring, and evaluation reports

Performance measure information is readily available in written periodic reports or bulletins that pulls together and analyses critical health information from all subsystems

Senior managers and policymakers demand complete, timely, accurate, relevant and validated HIS information

Policy and decision makers regularly use performance measure information to evaluate performance and set policies on health.

Data visualizations are widely used to display information at subnational health administrative offices and health facilities. They are up to date and clearly understood

Performance measurement information is demonstrably used in the national planning and in the resource-allocation processes (e.g., for annual integrated development plans, medium-term expenditure frameworks, long-term strategic plans, and annual health sector reviews)

Performance measure information is widely used, by sub-national management teams to set resource allocation in the annual budget processes

Performance measure information is used to advocate for equity and increased resources to disadvantaged groups and communities

Managers at all levels use health information for health service delivery management, continuous monitoring, and periodic evaluation

Section 9- Big Picture

9.1. How would you characterize the use of performance health information in Lebanon? All in all, do you think there is too much/not enough emphasis on performance measures?

9.2. What are the challenges that hinder the use of performance measurement information to inform health policymaking and decision-making processes?

9.3. Do you have suggestions on how to improve the role of performance measure information in informing the policymaking process?

Relevance

Quality

Timeliness/Accessibility

Analysis and interpretation

Format

9.4. Can you suggest up to three policies/ strategies/mechanisms that can help improve your institution's capacity to use data in policy and program development and implementation?

Malawi – Interview guide for Decision Makers

Participant details

Facilitator's Name: ____________________Date of the interview ____________

Name of the Facility:_________________ Location _____________

Respondent's age__________________ Respondent's Sex_________

Respondent's Position _______________ Qualification_____________

Respondent's years of experience__________________________

Introduction

What is your role in this organization?

What is your qualification

How long have you worked in this organization?

How long have you worked at your current position?

What are the main priorities of MOH in PHC?

What are your PHC priorities?

Currently, how can you rate your impact in PHC? Is your performance improving or not? How do you know?

Knowledge about data collection and use

Do you know how PHC data is collected at your institution?

If yes, may you briefly explain how it is done?

Do you think PHC data is collected in honest and effective manner?

Why is data collection and use important in your organization?

How is data utilized in policy formulation?

Performance Measurement

Please describe the ways in which the performance of PHC at your level is evaluated? Specifically, let us look at

Who does the evaluation?

When did the evaluation happen?

What data was used to assess the team's performance

How frequently is this done

When the performance assessment is complete, what type of feedback do you receive (oral or written)

How frequently do you receive the feedback about your performance?

What do you do with the feedback you receive about the PHC performance? May you give an example of a recent situation in which you used the performance results in your daily practice and services

Probe on: a. planning b. budgeting c. policy formulation

Do you have any specific example of a situation in which PHC performance results did not help you?

What do you think are the challenges associated with performance measurement?

What do you think can be done to address the challenges?

Factors affecting data driven decision making (DDDM)

May you briefly explain the things you consider when making a decision in your organization?

Probe on: data consultation, political factors, group/personal influence etc

Why are these things important in decision making?

Have you ever made important decision without relying on the available data?

If yes, why was data not relevant for that decision?

If not, why is relying on data important?

Are there factors/reasons for making a decision without relying on data?

Probe on: lack of data authenticity/trustworthiness, political influence, lack of understanding of data, lack of time to study the data, negligence etc

What do you think are the challenges associated with data driven decision making?

What do you think can be done to address the challenges?

Theme 3: Policies on data driven decision making

Are there policies that guide managers on data driven decision making?

If not, what drives managers to make a decision by relying on data?

Are all policies in PHC made in consultation with data?

If not, why?

What could be the result of formulating a policy without consulting data?

What does the ministry do?

Challenges and recommendations

Do you have any challenges on data collection and use in your organizations?

If yes, what are the challenges?

Probe on: lack of understanding of the data, bulkiness of the data, lack of time to study the data, lack of data authenticity, political interference etc

What do you think should be done to improve data collection and use in your organization?

Probe on: a. what should the data collectors and users do?

b. what should the government do?

Thank you very much. This is the end of our discussions.

Munar W, *et al. BMJ Open* 2022; 12:e060503. doi: 10.1136/bmjopen-2021-060503
