# Supplementary material for: Characterising performance information use in the primary healthcare systems of El Salvador, Lebanon and Malawi: multiple qualitative case study protocol
Source: BMJ Open. 2022 Nov 21;12(11):e060503. doi: 10.1136/bmjopen-2021-060503 (PMC9680164; doi:10.1136/bmjopen-2021-060503)
Supplement: Author's manuscript [file bmjopen-2021-060503.draft_revisions.pdf]

BMJ Open is committed to open peer review. As part of this commitment we make the peer review history of every article we publish publicly available.

When an article is published we post the peer reviewers' comments and the authors' responses online. We also post the versions of the paper that were used during peer review. These are the versions that the peer review comments apply to.

The versions of the paper that follow are the versions that were submitted during the peer review process. They are not the versions of record or the final published versions. They should not be cited or distributed as the published version of this manuscript.

BMJ Open is an open access journal and the full, final, typeset and author-corrected version of record of the manuscript is available on our site with no access controls, subscription charges or pay-per-view fees (http://bmjopen.bmj.com).

If you have any questions on BMJ Open's open peer review process please email info.bmjopen@bmj.com

BMJ Open

Characterising performance information use in the primary health care systems of El Salvador, Lebanon, and Malawi: Multiple case study protocol

Journal:	*BMJ Open*
Manuscript ID	bmjopen-2021-060503
Article Type:	Protocol
Date Submitted by the Author:	23-Dec-2021

Complete List of Authors:	Munar, Wolfgang; George Washington University, Global Health<br>Wahid, Syed; George Washington University, Division of Global Mental Health<br>Makwero, Martha; University of Malawi College of Medicine, school mof public health and Family medicine; Queen Elizabeth Central Hospital, Obstetrics and Gynaecology<br>El-Jardali, Fadi; Department of Health Management and Policy, Faculty of Health Sciences, American University of Beirut, Beirut, Lebanon, and 2Sch,<br>Dullie, Luckson; Partners In Health,<br>Yang, Wen-Chien; The George Washington University Milken Institute of Public Health, Global Health

Keywords:	Organisation of health services < HEALTH SERVICES ADMINISTRATION & MANAGEMENT, International health services < HEALTH SERVICES ADMINISTRATION & MANAGEMENT, Health policy < HEALTH SERVICES ADMINISTRATION & MANAGEMENT

SCHOLARONE™
Manuscripts

Characterising performance information use in the primary health care systems of El Salvador, Lebanon, and Malawi: Multiple case study protocol

Author list:

Wolfgang Munar, George Washington University, Milken Institute School of Public Health, Washington, DC, United States of America

Syed S. Wahid, George Washington University, Milken Institute School of Public Health, Washington, DC, United States of America

Martha Makwero, Kamuzu University of Health Sciences, School of Medicine and Dentistry, Blantyre, Malawi

Fadi Al-Jardali, American University of Beirut Faculty of Health Sciences, Beirut, Lebanon

Luckson Dullie, Partners in Health, Malawi

Wen-Chien Yang, George Washington University, Milken Institute School of Public Health, Washington, DC, United States of America

Corresponding author:

Wolfgang Munar
Milken Institute School of Public Health
950 New Hampshire Avenue NW
Washington DC 20552, United States of America

Email: wolfgangmunar@gwu.edu

Phone: +1.206.218.7298 (Mobile) +1.202.994.1015 (Office)

Keywords

Performance evaluation; Performance measurement and management; Performance information; Organisation and management of care; Decision making

Wordcount: 3,946, including abstract

For peer review only - http://bmjopen.bmj.com/site/about/guidelines.xhtml

ABSTRACT

Introduction. Governments in low- and middle-income countries (LMICs) and official development assistance agencies use a variety of performance measurement and management (PMM) approaches to improve the performance of healthcare systems. The effectiveness of such approaches is contingent on the extent to which managers and care providers use performance information or not. To date, major knowledge gaps exist about the contextual factors that contribute, or not, to performance information use by primary healthcare (PHC) decisionmakers in LMICs. This study addresses three research questions: 1) How do decision makers use performance information, and for what purposes? 2) What are the contextual factors that influence the use or non-use of performance information? and 3) What are the proximal outcomes reported by PHC decisionmakers from performance information use?

Methods and analysis. We present a theory-driven, qualitative study with a multiple case study design to be conducted in El Salvador, Lebanon, and Malawi. The study entails semi structured interviews and document review. Interviews will be conducted with approximately sixty respondents including PHC system decisionmakers and providers. We follow an interdisciplinary theoretical framework that draws on health policy and systems research, public administration, organisational science, and health services literature. Data will be analysed using thematic analysis to explore how respondents use performance information or not, and for what purposes as well as their experienced barriers and facilitators.

Ethics and dissemination. The ethical boards of the participating universities (anonymised) approved the protocol presented here. Study results will be disseminated through peer-reviewed journals and global health conferences.

For peer review only - http://bmjopen.bmj.com/site/about/guidelines.xhtml

Strengths and limitations of this study

- The study described here follows an interdisciplinary framework that helped inform the design of a multiple case study in El Salvador, Lebanon and Malawi.
- The research will provide context-specific descriptions of performance information use and non-use. The evidence generated will be used to refine the theoretical framework for future evaluations of performance information use.
- The use of a case study design with embedded units in different country contexts can contribute to analytic generalizations about the influence of contextual factors on performance information use and non-use.
- Study limitations include a degree of desirability bias in semi-structured interviews and potential loss of rapport with respondents due to the use of virtual interviews.

For peer review only

For peer review only - http://bmjopen.bmj.com/site/about/guidelines.xhtml

I. Introduction

PHC systems are first points of entry into health service delivery, are essential for people-centred service delivery, and connect citizens to health systems (1). High-performing PHC systems are also central elements in the preparedness for and response to pandemics and other public health emergencies (2, 3).

During the last forty years, PMM systems have become prevalent in healthcare management and organisation (4-6). Governments, official development assistance agencies, and various global health partnerships have used diverse PMM approaches to improve performance of policies and programs in maternal and child health (7, 8), HIV/AIDS, malaria, and tuberculosis (9), and other global health priorities. Outcomes-driven financing approaches have also been used as a means to improving PHC system performance (10).

Performance measurement and management systems

PMM systems were originally conceived as ensembles of management control mechanisms designed to stimulate the delivery of organisational priorities and influencing desirable organisational behaviours (11-13). However, depending on contextual factors and historical antecedents, PMM systems have evolved in response to contrasting organisational logics (14). *Directive* systems tend to be guided by a logic of consequences, are prevalent in systems that favour audit cultures (15), are designed with a view towards accountability, and follow the utility-maximising assumptions of *Homo economicus* in agency theory (16). *Enabling* approaches, on the other hand, are guided by logics of improvement and learning; can create conditions for adaptive and iterative cycles of error, reflection, sensemaking, and corrective action; and conceive of performance as emergent processes, influenced by managers and workers' agency, motives, means, and opportunities (17, 18).

For peer review only - http://bmjopen.bmj.com/site/about/guidelines.xhtml

Studies on PMM systems' effectiveness have identified several sources of leverage for performance improvement in public sector organisations (19-22). Organisational performance tends to be positively associated with PMM systems that reinforce workforce motivation (23); promote performance measurement at multiple levels (i.e., individual, interpersonal, and inter-organisational) (24); and where decisionmakers use of the information generated through performance appraisals (25, 26).

Governments use and official development assistance agencies promote a diverse set of approaches to performance management including multifaceted financial arrangements, accountability approaches, and implementation strategies (27). An evidence gap map of PMM interventions in the PHC systems of LMICs showed that most primary studies to date have focused on provider-level implementation strategies such as in-service training and supervision, and on financial arrangements like pay-for-performance (28). The mapping exercise also identified absolute gaps in evidence for PMM interventions that operate at organisational levels, particularly accountability arrangements like public release of performance information or social accountability. There is also limited knowledge about the role of contextual factors in enabling or hindering the use of performance information at the organisational level of teams, facilities, and district health systems. Table 1 summarises the interventions mapped in the evidence gap map above.

The widespread use of PMM systems in the public sector, particularly in health, has shown that, when not tailored to context, PMM systems can not only be ineffective but can also contribute to negative outcomes such as gaming, goal displacement, and data manipulation (29). Further, public administration research has also shown that decision makers do not consistently use performance information and that, when they do, the largest impacts on service delivery are attained when it is used as part of organisational dialogues that inform changes in operational and strategic direction (25, 26, 30). The literature has also shown that official development

For peer review only - http://bmjopen.bmj.com/site/about/guidelines.xhtml

For peer review only

assistance agencies promote and use various PMM approaches for improving accountability to donors and beneficiaries; enhancing organisational learning and communications; and informing changes in strategic direction (31).

Table 1 - Performance measurement and management interventions for the organisation and delivery of healthcare

**Implementation strategies**	**Accountability arrangements**	**Financial arrangements**
Provider-level: Clinical practice guidelines, reminders, in-service training, and continuous education<br>Organisational level: Clinical incident reporting; clinical practice guidelines; local opinion leaders; continuous quality improvement; and supervision	Individual- or organisational-level: Audit and feedback<br>Community-level: Public release of performance information, social accountability	Individual and organisational level: Results-Based Financing, Pay for Performance, and other provider incentives and rewards.

The literature on routine health information systems (RHIS) in LMICs has identified organizational, behavioural, and technical challenges to the production and use of information including, among others, fragmentation, duplication, and poor data quality (32). It has also been shown that even when quality health information is available, LMIC health managers may not use it, leading to suboptimal decision-making processes that may negatively affect governance and healthcare management. Previous research has also found that non-use of data from RHIS can be explained by lack of motivation or scarce capacity among decision makers; and by non-existing or poorly functioning feedback and supervision mechanisms (33-36).

II. Methods and analysis

Study aims and research questions

The study described here will assess the experiences of PHC decisionmakers and providers with performance measurement and management in El Salvador, Lebanon, and Malawi. Research findings will be used to inform an applied research agenda on PHC system

For peer review only - http://bmjopen.bmj.com/site/about/guidelines.xhtml

performance; contribute to improve the measurement and management of PHC systems performance; and develop an evaluation framework for assessing performance information use in other country contexts. Our research questions are: 1) How do PHC system decision makers use performance information, or not, and for what purposes? 2) What are the contextual factors that influence the decision to use performance information, or not? 3) What are the proximal outcomes reported by PHC decision makers from performance information use and non-use?

Theoretical framework

Based on PMM models in public administration research, implementation research, and organisational science (5, 21, 37-39) we developed an interdisciplinary theoretical framework to help guide study design. PMM systems are conceptualised as continuous and recursive cycles of 1) organisational priorities and goals; 2) incentives; 3) performance measurement, feedback, and sense-making; 4) implementation strategies; and 5) performance outcomes (5), as represented in figure 1.

Figure 1- Performance measurement and management model

Organisational priorities and goals are the ultimate expression of what desirable performance ought to be; they are identifiable in policy documents, summarized in logical models, and sometimes reflected as measurable targets in performance frameworks. Incentive systems are managerial practices aimed at stimulating workforce motivation and fostering organisational performance by means of extrinsic and intrinsic stimuli. Extrinsic motivators include rewards, recognition, pay for performance, bonuses, and in-kind incentives, among others (40). Intrinsic motivators aim to trigger satisfaction of workers' basic needs such as competence, autonomy, and connection (41, 42). It is believed that both types of motivators are central to organisational performance (43).

For peer review only - http://bmjopen.bmj.com/site/about/guidelines.xhtml

For peer review only

Performance measurement processes generate raw data about past performance and use metrics that reflect organisational priorities and goals. Performance data are usually compiled into registers that feed into routine health information systems (RHIS), and can be summarised and disseminates via reports, scorecards, and dashboards. Given the perceived low-quality of RHIS, particularly in LMICs (34), performance data is also sourced from cross-sectional population surveys. The latter have become one of the most frequently used data sources for tracking health programs' performance (35, 36).

The data acquired via RHIS and/or population surveys are usually contrasted against expected targets and goals which, in turn, are disseminated in ways that generate performance information flows aimed at different users. Upward flows bring information through organisational hierarchies usually for reporting and accountability purposes. Information can also be fed-back to the frontlines of service provision as part of feedback and audit, quality improvement, or supportive supervision processes (44). As organisational actors engage with performance data, ascribe meaning to it, and imagine future courses of action in response to perceived gaps in performance, the managerial processes above can contribute to collective sensemaking (45), a process that helps people 'understand issues or events that are novel, ambiguous, confusing or in some other way violate expectations' (46). It can also inform decisions among organisational actors to engage or not in addressing the gaps in performance made evident by available information. Action plans, budgets, changes in service delivery, and other processes of course-correction can thus be considered for future implementation.

Once courses of action are decided, organisational actors can deploy various strategies to implement them. Implementation strategies help system actors appraise and respond in adaptive fashion to factors in their immediate environment that can enable or hinder collective action (see table 1). In the short term, performance information can be used for planning, compliance, reporting, or rapid course-correction purposes, among others; it can also be

For peer review only - http://bmjopen.bmj.com/site/about/guidelines.xhtml

misused through gaming processes, or not used (29). As iterative PMM cycles are repeated through time, performance information can also be used (or not) as the basis for testing new processes and services, for internal advocacy, and/or for policy formation.

PMM cycles can contribute to proximal performance outcomes that feed into long causal chains of outcomes occurring at multiple levels within an organisation (e.g., at individual, team, and organisational levels). Outcomes can include 1) proximal changes resulting from using performance information (or not), such as action plans implemented, compliance with procedural standards, timely reporting, and rapid course-correction; 2) intermediate effects emerge at the organisational behaviour level, and may include changes in workforce motivation, job satisfaction, morale, or organisational commitment; and 3) downstream population-level health effects and equity outcomes resulting from the iterative repetition of PMM cycles in dynamic and changing environments.

We integrated the elements of the PMM model described above into a theoretical framework that represents the hypothetical process of performance change and the role played by performance information use and non-use. The framework contains the following elements: external context; PMM approaches in use within the public sector; performance information production and use; PHC systems' internal organisational environment; and the causal pathways connecting performance information use and non-use to proximal, intermediate, and distal outcomes.

The theoretical framework is represented in figure 2. Here, the managerial practices used to measure and change performance are influenced by external and internal contextual factors and by the implementation strategies in use, and modulated by the use and non-use of performance information. The processes of change thus generated can contribute, via long causal chains, to a variety of outcomes and impacts. Proximal effects from performance information use are represented by single-loop learning effects (47) such as changes in planned action, rapid course-

For peer review only - http://bmjopen.bmj.com/site/about/guidelines.xhtml

For peer review only

correction, and improvements in service quality. The repetition of such iterative cycles may, in turn, contribute to the emergence of second-loop learning effects such as changes in strategic direction and new practices among service providers and managers (48).

Figure 2 – Theoretical framework

The use of performance information is causally linked to proximal performance outcomes at the individual level of providers and patients; those outcomes are also causally connected to intermediate outcomes at the organisational level such as improved workforce motivation, enhanced organizational commitment, increased trust between providers and PHC system users, and reduced staff turnover, among others. These outcomes can contribute to distal population health and equity outcomes (intended and otherwise). Depending on context, the causal chain of outcomes described above can also be interrupted, be limited to isolated pockets of excellence, or be altogether absent.

Study design

The present study will explore the uses of performance information in the PHC systems in El Salvador, Lebanon, and Malawi. Investigation across different contexts allows for the generation of context-specific insights of value to local actors and, potentially, to broader understandings of the phenomena of interest (49).

To address the research questions, we chose a theory informed, multiple case study design with embedded units of analysis (50). Case studies are well-suited for obtaining an in-depth understanding of context-specific processes in complex systems (51). Here, a case is defined as each country's PMM practices; the two units of analysis included are PHC service provision and PHC policy implementation at national and subnational levels.

For peer review only - http://bmjopen.bmj.com/site/about/guidelines.xhtml

Study setting

El Salvador

El Salvador is a lower-middle income country with a population of 6.4 million. Since the conclusion in 1992 of a civil war, the country reduced inequality by about 5 percentage points between 2007 and 2016; increased coverage of institutional deliveries and immunisation to 98% and 93%, respectively; and achieved the under-five mortality reduction for the Millennium Development Goals (52, 53).

Starting in 2009, El Salvador universalised access to free, comprehensive PHC. Existing infrastructure was reorganised into PHC networks, one for each of the Departments in which the country is administratively divided. Service delivery was delegated to multi- professional teams of PHC providers. The oversight of each Departments' network is the responsibility of a decentralised MOH coordination team called SEBASI in its Spanish acronym. PHC teams have a nominal catchment area of 3,000 individuals and are co-located within the communities they serve. A basic PHC team is made up of one medical doctor, two nurses, and up to three community health promoters; some teams have specialised care providers. PHC teams provide community outreach as well as facility-based services and deliver a package of benefits containing approximately 300 interventions (54).

In 2011, the government of El Salvador joined the Salud Mesoamerica Initiative (SMI), a public-private partnership focused on improving the performance of PHC systems in the eight nation states of Mesoamerica. In El Salvador, SMI operates in 75 PHC teams operating in the poorest rural municipalities in the country. PMM interventions used include PHC team target-setting; monitoring of PHC teams' performance using population and facility surveys and RHIS; provision of feedback to teams; and team-based in-kind incentives (54, 55).

For peer review only - http://bmjopen.bmj.com/site/about/guidelines.xhtml

Lebanon

Lebanon is home to approximately 6,8 million people and is classified as an upper-middle income country (56). However, the financial crisis that started in 2019 reduced real per-capita gross domestic product 37.1 percent between 2018 and 2021 The country also hosts the largest number of refugees per capita in the world (57) and has suffered additional internal shocks. The combined effect of these various shocks has put major pressure on an already stretched healthcare system (58, 59).

PHC services in Lebanon are provided by a combination of private-for-profit and not-for-profit providers; the latter are the most accessible and used sources of care by vulnerable Lebanese and refugee populations (60, 61). Lebanon's official PHC network is comprised of 213 centres that have contractual agreements with the Ministry of Public Health based on pre-met community care delivery standards.

In terms of performance measurement at the PHC level, the MOH has developed policies and practices to monitor service delivery patterns, quality of care and performance of PHC centres within the national network (62). Monitoring involves regular visits by MOH inspectors and administration of patient satisfaction surveys (62). Accreditation is also used to regulate the quality of care at the PHC level. By establishing a National Accreditation Program for PHC centres in 2009, the MOH aimed to ensure continuous and sustainable quality control, improve compliance with legal and safety standards, enhance transparency and accountability, and establish a positive image of standards of practice and service at PHC centres (62).

Despite the various health reforms implemented in Lebanon, there is still no active national strategic plan designed around PHC (63-66). Furthermore, many PHC centres remain underdeveloped with no availability of basic diagnostic imaging and laboratory medicine, resulting in perceived lack of confidence on the quality of services offered (59).

For peer review only - http://bmjopen.bmj.com/site/about/guidelines.xhtml

Malawi

Malawi is a land-locked, low-income country with a population of approximately 18.6 million. The economy is mainly dependent on the agricultural sector which employs 80% of the population. A five-year development plan, Malawi's Growth and Development Strategy, guides the country's development; the current plan is focused on education, health, agriculture, energy, and tourism (67).

Malawi's epidemiological profile combines high burden of disease from both preventable conditions as well as noncommunicable diseases. The country has a high population density and a total fertility rate of 4.4. Prevalent social determinants of health include poverty and inequality, high levels of illiteracy and limited coverage of social safety programs (67, 68).

Primary care is the main platform for the delivery of health services in Malawi. However, the PHC system is characterised by poor distribution of human and physical resources, fragmentation of services and chronic shortages of staff (69). In an effort to reduce service fragmentation, Malawi developed in 2017 a new community health policy centred on a team-based approach. Community health teams (CHT) comprise health surveillance assistants (HSAs), clinicians, environmental health officers and community health volunteers (70).

Data collection

In the research presented here will utilize document review and semi-structured interviews with informants with 'great knowledge…who can shed light on the inquiry issues' (71). We will use document review to identify domestic priorities and explore the external context, available resources, ongoing official development assistance programs. Documents to be reviewed include MOH policy documents, strategic frameworks, operational plans, results frameworks, performance reports, and logical models, among others. Semi-structured interviews will be conducted with PHC decisionmakers at the national and subnational levels and with providers.

For peer review only - http://bmjopen.bmj.com/site/about/guidelines.xhtml

To be eligible for inclusion, decisionmakers will be current or former officials responsible for PHC system policy formulation or implementation at national and subnational levels; providers will be staff currently working as clinical care providers or community health workers.

Respondent selection and recruitment will follow an information power approach based on criteria that are suitable for reaching saturation in qualitative studies using non-probabilistic, purposive sampling (72). We will design respondent sampling guided by our understanding of the types of participants that can provide highly specific information to address the study's research questions; insights from the preliminary theoretical framework; and responsive to the quality of the dialogue elicited during data collection. The estimated number of respondents to be interviews in the three countries is 60 respondents. Respondent inclusion criteria will be calibrated to the context of each study setting; site-specific approaches to data collection will be reported in each country case study.

In the interviews with service providers, we will explore experiences about the PHC system organisational environment; the ways in which PHC performance is measured, analysed, and made sense of; the extent to which performance information is used or not, and for what purposes, and the reported effects from using performance information. Interviews with decisionmakers at national and subnational levels will explore PHC priorities, goals and/or targets; characterise the public sector institutional context; explore sources and frequency of performance data appraisal; and inquire about the uses of performance information. We will also triangulate the data resulting from document review and interviews, and the experiences reported PHC by the two types of respondents.

In each country, the research team will develop a Project Brief summarizing the study's aims and highlighting the voluntary nature of participation. An invitation to participate in the interview will be sent individually via e-mail to each potential respondent. Once the respondent agrees to

For peer review only - http://bmjopen.bmj.com/site/about/guidelines.xhtml

participate in the interview, a remote interview will be scheduled (or in-person, if allowed by ethical review board). Before initiation of the interview, the interviewer shall read the consent form and obtain verbal consent from the interviewee which will be recorded and reflected in the interview transcript accordingly. Site-specific interview guidelines are available in the Supplementary file.

Analysis

Interviews will be audio taped, transcribed verbatim and imported into NVivo 12.0. Transcripts will be coded independently by at least two researchers in each country. We will use an iterative, directed approach to analysis (73) informed by the theoretical framework. The latter shall also inform the design of a codebook to guide deductive coding of the data. Inductive codes emerging from the data will also be identified and included in the analysis. We will convene analytic workshops among the research teams in participating countries to discuss the codebook, the coding process, thematic analysis, and data synthesis procedures.

After the conclusion of coding in each country, we will execute code queries for each code, stratified by respondent type (e.g., providers and decision makers) to extract code-specific data. Subsequently, we will review and summarize the code-specific and respondent-specific data from the query outputs into code summary memos using a standardized template.

Code summary memos will include a respondents table to capture brief and relevant information from each type of respondent, and narratives constructed by the researcher reviewing the query output, supported by exemplary quotes. Code summary memos will include deviant narratives and quotes that run counter to the main narrative(s) and a section for recording researcher insights on where and how codes may be connected to others. In a final step, the synthesized data in the code summary memos will be organized into thematic matrices to formalize linkages between codes and construct themes. The resulting themes will be used to report country-

For peer review only - http://bmjopen.bmj.com/site/about/guidelines.xhtml

specific findings and to develop a refined theoretical framework. Results for each country case will be organised using the COREQ checklist (74) and will be disseminated through symposia and peer-reviewed journals.

Study limitations include a degree of desirability bias in semi-structured interviews and potential loss of rapport with respondents due to the use of virtual interviews.

Patient and public involvement

Neither patients nor public were involved in the conduct, reporting or dissemination of the research presented in this protocol.

III. Ethical considerations

The ethical approval for this study was provided by the Institutional Review Boards of the following universities (anonymized). We will follow ethical principles of voluntary and informed involvement in the study, confidentiality, and safety of all participants. Verbal consent will be obtained from all respondents and be reflected in the respective interview transcripts.

A database will be maintained containing information on all interviews completed, including demographic data and time of the interview as well as confirming verbal consent by each respondent. All identifying information will be stored in an encrypted database, hosted in encrypted and password protected cloud services provided by each of the hosting research institutions. The identifier information database will be permanently deleted after the completion of data analysis.

For peer review only - http://bmjopen.bmj.com/site/about/guidelines.xhtml

IV. Study significance

Research on the use of performance information in PHC systems is scarce; multi-country case studies in LMICs are inexistent to the best of our knowledge. The study presented here can contribute to an understanding of the contextual factors and organisational environments that enable or hinder the use of performance information in the PHC systems of El Salvador, Lebanon, and Malawi. Such knowledge can inform future research and contribute to improve the strategies used in LMIC settings to measure and manage PHC system performance.

Author's contributions

The study was conceptualised by Wolfgang Munar, Martha Makwero and Fadi Al-Jardali. The first draft was written by Syed Wahid, Wen-Chien Yang, and Wolfgang Munar, with inputs from Luckson Dullie, Martha Makwero and Fadi Al-Jardali. All authors have read and approved the final manuscript.

Acknowledgement

We would like to acknowledge the Primary Health Care Research Consortium and for their support, in particular Doctor D. Praveen and Manushi Sharma. This work was supported, in whole, by the Bill & Melinda Gates Foundation [INV-000970]. Under the grant conditions of the Foundation, a Creative Commons Attribution 4.0 Generic License has already been assigned to the Author Accepted Manuscript version that might arise from this submission.

Funding Statement

This study is funded by the Bill and Melinda Gates Foundation through the Primary Health Care Research Consortium hosted at the George Institute for Global Health in India [INV-000970]

Declaration of Interests

The researchers declare that they have no conflicts of interest.

For peer review only - http://bmjopen.bmj.com/site/about/guidelines.xhtml

V. References

1. WHO. The world health report 2008: primary health care (Now more than ever). Geneva: World Health Organization; 2008.

2. Peiris D, Sharma M, Praveen D, Bitton A, Bresick G, Coffman M, et al. Strengthening primary health care in the COVID-19 era: a review of best practices to inform health system responses in low- and middle-income countries. WHO South-East Asia Journal of Public Health. 2021;10(3):6-25.

3. Friedrich M. Declaration of Astana emphasizes primary care as key to world health. Jama. 2018;320(23):2412-.

4. Murray CJ, Frenk J. A framework for assessing the performance of health systems. Bulletin of the world Health Organization. 2000;78:717-31.

5. Pollitt C. Performance management 40 years on: a review. Some key decisions and consequences. Public Money & Management. 2018;38(3):167-74.

6. OECD. Measuring up: improving health system performance in OECD countries. Paris: OECD Publishing; 2002.

7. Fernandes G, Sridhar D. World Bank and the global financing facility. BMJ. 2017;358.

8. Eichler R, Agarwal K, Askew I, Iriarte E, Morgan L, Watson J. Performance-based Incentives to Improve Health Status of Mothers and Newborns: What Does the Evidence Show? Journal of Health Population and Nutrition. 2013;31(4):36-47.

9. Fan V, Denizhan Duran R. Grant Performance and Payments at the Global Fund. Washington, DC: Center for Global Development; 2013. Contract No.: 31.

10. Mokdad AH, Palmisano EB, Zuniga-Brenes P, Rios-Zertuche D, Johanns CK, Schaefer A, et al. Supply-side interventions to improve health: Findings from the Salud Mesoamerica Initiative. Plos One. 2018;13(4).

11. Broadbent J, Laughlin R. Performance management systems: A conceptual model. Manage Account Res. 2009;20(4):283-95.

12. Ferreira A, Otley D. The design and use of performance management systems: An extended framework for analysis. Manage Account Res. 2009;20(4):263-82.

13. Talbot C. Theories of performance: Organizational and service improvement in the public domain. New York, NY: Oxford University Press; 2010.

14. Pollitt C. The logics of performance management. Evaluation-Us. 2013;19(4):346-63.

15. Shore C, Wright S, Amit V, Brown J, Bruun Jensen C, Maguire M, et al. Audit culture revisited: Rankings, ratings, and the reassembling of society. Current Anthropology. 2015;56(3):431-2.

16. Eisenhardt KM. Agency theory: An assessment and review. Academy of Management Review. 1989;14(1):57-74.

17. Newton-Lewis T, Munar W, Chanturidze T. Performance management in complex adaptive systems: a conceptual framework for health systems. BMJ Global Health. 2021;6(7):e005582.

18. Davis JH, Schoorman FD, Donaldson L. Toward a stewardship theory of management. Academy of Management Review. 1997;22(1):20-47.

For peer review only - http://bmjopen.bmj.com/site/about/guidelines.xhtml

19. Walker RM, Lee MJ, James O, Ho SMY. Analyzing the Complexity of Performance Information Use: Experiments with Stakeholders to Disaggregate Dimensions of Performance, Data Sources, and Data Types. Public Administration Review. 2018;78(6):852-63.

20. Walker RM, Boyne GA, Brewer GA, editors. Public management and performance: Research directions. Cambridge, UK: Cambridge University Press; 2012.

21. Franco-Santos M, Lucianetti L, Bourne M. Contemporary performance measurement systems: A review of their consequences and a framework for research. Manage Account Res. 2012;23(2):79-119.

22. Levesque J-F, Sutherland K. What role does performance information play in securing improvement in healthcare? A conceptual framework for levers of change. BMJ open. 2017;7(8):e014825.

23. Kanfer R, Frese M, Johnson RE. Motivation Related to Work: A Century of Progress. Journal of Applied Psychology. 2017;102(3):338-55.

24. Van Dooren W, Bouckaert G, Halligan J, editors. Performance measurement in the Public Sector. Second ed: Routledge; 2015.

25. Pandey SK. Performance Information Use: Making Progress But a Long Way to Go. Public Perform Manag. 2015;39(1):1-6.

26. Moynihan DP, Pandey SK. The Big Question for Performance Management: Why Do Managers Use Performance Information? Journal of Public Administration Research and Theory. 2010;20(4):849-66.

27. EPOC. Effective Practice and Organisation of Care (EPOC). EPOC taxonomy Cochrane Collaboration. Norwegian Satellite2015 [cited 2019 October 17, 2019]. Available from: https://epoc.cochrane.org/epoc-taxonomy.

28. Munar W, Snilstveit B, Aranda LE, Biswas N, Baffour T, Stevenson J. Evidence gap map of performance measurement and management in primary healthcare systems in low-income and middle-income countries. BMJ Global Health. 2019;4(Suppl 8):e001451.

29. Franco-Santos M, Otley D. Reviewing and Theorizing the Unintended Consequences of Performance Management Systems. International Journal of Management Reviews. 2018;20(3):696-730.

30. Moynihan DP. The dynamics of performance management: Constructing information and reform. 12 ed. Washington, DC: Georgetown University Press; 2008 March 11, 2008. 224 p.

31. Vahamaki J, Verger C. Learning from Results-Based Management evaluations and reviews. Paris: OECD; 2019.

32. Leon N, Balakrishna Y, Hohlfeld A, Odendaal WA, Schmidt B-M, Zweigenthal V, et al. Routine Health Information System (RHIS) improvements for strengthened health system management. Cochrane Database of Systematic Reviews. 2020(8).

33. Aqil A, Lippeveld T, Hozumi D. PRISM framework: a paradigm shift for designing, strengthening and evaluating routine health information systems. Health Policy and Planning. 2009;24(3):217-28.

34. Hotchkiss DR, Aqil A, Lippeveld T, Mukooyo E. Evaluation of the Performance of Routine Information System Management (PRISM) framework: evidence from Uganda. BMC Health Services Research. 2010;10.

For peer review only - http://bmjopen.bmj.com/site/about/guidelines.xhtml

35. Wagenaar BH, Hirschhorn LR, Henley C, Gremu A, Sindano N, Chilengi R, et al. Data-driven quality improvement in low-and middle-income country health systems: lessons from seven years of implementation experience across Mozambique, Rwanda, and Zambia. Bmc Health Services Research. 2017;17.

36. Hoxha K, Hung YW, Irwin BR, Grépin KA. Understanding the challenges associated with the use of data from routine health information systems in low-and middle-income countries: A systematic review. Health Information Management Journal. 2020:1833358320928729.

37. Contandriopoulos D, Champagne F, Denis J-L. The multiple causal pathways between performance measures' use and effects. Med Care Res Rev. 2014;71(1):3-20.

38. Contandriopoulos D, Lemire M, Denis JL, Tremblay E. Knowledge Exchange Processes in Organizations and Policy Arenas: A Narrative Systematic Review of the Literature. Milbank Quarterly. 2010;88(4):444-83.

39. Damschroder LJ, Aron DC, Keith RE, Kirsh SR, Alexander JA, Lowery JC. Fostering implementation of health services research findings into practice: a consolidated framework for advancing implementation science. Implementation Science. 2009;4(1):1-15.

40. Heinrich CJ, Marschke G. Incentives and Their Dynamics in Public Sector Performance Management Systems. J Policy Anal Manag. 2010;29(1):183-208.

41. Deci EL, Olafsen AH, Ryan RM. Self-Determination Theory in Work Organizations: The State of a Science. Annual Review of Organizational Psychology and Organizational Behavior, Vol 4. 2017;4:19-43.

42. Gagne M, Deci EL. Self-determination theory and work motivation. Journal of Organizational Behavior. 2005;26(4):331-62.

43. Cerasoli CP, Nicklin JM, Ford MT. Intrinsic Motivation and Extrinsic Incentives Jointly Predict Performance: A 40-Year Meta-Analysis. Psychological Bulletin. 2014;140(4):980-1008.

44. Colquhoun HL, Carroll K, Eva KW, Grimshaw JM, Ivers N, Michie S, et al. Advancing the literature on designing audit and feedback interventions: identifying theory-informed hypotheses. Implementation Science. 2017;12(1):117.

45. Weick KE, Sutcliffe KM, Obstfeld D. Organizing and the process of sensemaking. Organization Science. 2005;16(4):409-21.

46. Maitlis S, Christianson M. Sensemaking in organizations: Taking stock and moving forward. Academy of Management Annals. 2014;8(1):57-125.

47. Argyris C, Schon DA. Organizational learning: A theory of action perspective. Reis. 1997(77/78):345-8.

48. Argyris C. Double loop learning in organizations. Harvard Bus Rev. 1977;55(5):115-25.

49. Gilson L. Health policy and systems research: a methodology reader. Geneva: World Health Organization; 2012.

50. Yin RK. Applications of case study research. Third ed. Thousand Oaks, USA: Sage Publications; 2011 June 21, 2011. 264 p.

51. Yin RK. Case study research and applications: Design and methods. Thousand Oaks, CA.: Sage Publications; 2018.

52. World-Bank. The World Bank in El Salvador Washington, DC. USA2019 [Available from: https://www.worldbank.org/en/country/elsalvador/overview.

For peer review only - http://bmjopen.bmj.com/site/about/guidelines.xhtml

53. IADB. El Salvador Washington, DC. USA2019 [Available from: https://www.iadb.org/en/alianza-para-la-prosperidad/el-salvador.

54. Munar W, Wahid SS, Mookherji S, Innocenti C, Curry L. Team-and individual-level motivation in complex primary care system change: A realist evaluation of the Salud Mesoamerica Initiative in El Salvador. Gates Open Research. 2018;2.

55. Bernal P, Martinez S. In-kind incentives and health worker performance: Experimental evidence from El Salvador. Journal of Health Economics. 2020;70:102267.

56. World Bank. Lebanon Country Overview Washington, DC2020 [Available from: https://www.worldbank.org/en/country/lebanon/overview

57. UNHCR. Lebanon. Time-series Syrian Refugees Registered by Cadastral in Lebanon (2012-2019) 2019 [Available from: https://data2.unhcr.org/en/documents/details/40569

58. Harake W, Abou Hamde NM. So When Gravity Beckons, the Poor Don't Fall. Washington, DC: The World Bank; 2019. Contract No.: 145454.

59. Ismaeel H, El Jamal N, Dumit N, Al-Chaer E. Saving the Suffering Lebanese Healthcare Sector: Immediate Relief while Planning Reforms. Paris: Arab Reform Initiative; 2020.

60. Hamadeh RS, Kdouh O, Hammoud R, Leresche E, Leaning J. Working short and working long: can primary healthcare be protected as a public good in Lebanon today? Conflict and health. 2021;15(1):1-9.

61. Hemadeh R, Kdouh O, Hammoud R, Jaber T, Khalek LA. The primary healthcare network in Lebanon: a national facility assessment. Eastern Mediterranean Health Journal. 2020;26(6-2020):700.

62. El- Jardali F, Fadlallah R, Matar L. Primary health care systems (PRIMASYS): comprehensive case study from Lebanon. Geneva: World Health Organization; 2017.

63. Ministry of Public Health L. Health Response Strategy: Maintaining Health Security, Preserving Population Health & Saving Children and Women Lives A New Approach 2016 & Beyond Beirut, Lebanon2016 [Available from: https://www.moph.gov.lb/userfiles/files/Strategic%20Plans/HealthResponseStrategy-updated.pdf

64. Ministry of Public Health L. Health Strategic Plan: Strategic Plan for the medium term (2016 to 2020) Beirut, Lebanon: Ministry of Public Health, Lebanon; 2016 [Available from: https://www.moph.gov.lb/en/Pages/9/11666/.

65. Ministry of Public Health L. MOPH Strategy 2025 Beirut, Lebanon2018 [cited 2021. Available from: https://www.moph.gov.lb/en/view/19754/ministry-of-public-health-strategy-health-2025-

66. Ministry of Public Health L. Health Reform in Lebanon Beirut, Lebanon2021 [Available from: https://www.moph.gov.lb/en/view/1279/health-reform-in-lebanon

67. World Bank. Malawi. Overview Washington, DC2021 [cited 2021. Available from: https://www.worldbank.org/en/country/malawi/overview#1.

68. WHO. Malawi Geneva2021 [cited 2021. Available from: https://www.afro.who.int/countries/malawi.

69. Makwero MT. Delivery of primary health care in Malawi. African Journal of Primary Health Care and Family Medicine. 2018;10(1):1-3.

For peer review only - http://bmjopen.bmj.com/site/about/guidelines.xhtml

70. Angwenyi V, Aantjes C, Kondowe K, Mutchiyeni JZ, Kajumi M, Criel B, et al. Moving to a strong (er) community health system: analysing the role of community health volunteers in the new national community health strategy in Malawi. BMJ global health. 2018;3(Suppl 3):e000996.

71. Patton MQ. Qualitative research and evaluation methods. Fourth ed. Thousand Oaks, CA: Sage publications; 2014. 806 p.

72. Malterud K, Siersma VD, Guassora AD. Sample size in qualitative interview studies: guided by information power. Qual Health Res. 2015;26(13):1753-60.

73. Hsieh H-F, Shannon SE. Three approaches to qualitative content analysis. Qual Health Res. 2005;15(9):1277-88.

74. Tong A, Sainsbury P, Craig J. Consolidated criteria for reporting qualitative research (COREQ): a 32-item checklist for interviews and focus groups. International Journal for Quality in Health Care. 2007;19(6):349-57.

For peer review only

For peer review only - http://bmjopen.bmj.com/site/about/guidelines.xhtml

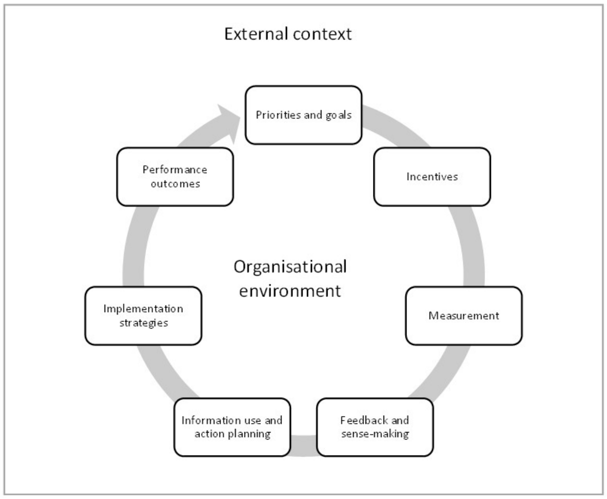

Figure 1 - Performance measurement and management model

338x190mm (96 x 96 DPI)

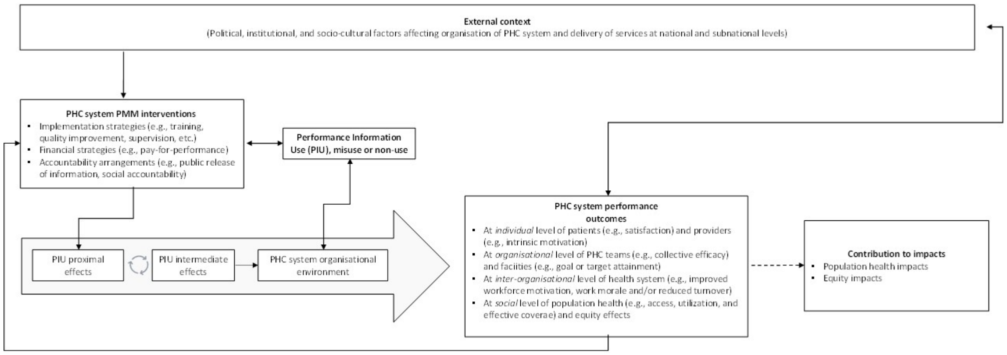

Figure 2 - Theoretical framework

338x190mm (96 x 96 DPI)

Supplementary files

For peer review only

El Salvador interview guidelines- PHC team members

Introduction.

For the past decade, the Salud Mesoamerica Initiative has been working in collaboration with the Ministry of Health to promote maternal and child health in El Salvador. The overall objective of this study is to assess the ways in which MCH performance information is collected and used at the local and national levels in El Salvador. The team obtained non-objection from the Ministry of Health to conduct this research. The findings will be used to develop better health care practices in El Salvador and hopefully contribute to better health outcomes for Salvadorans.

1. Do you recall participating (being interviewed) in 2018 for a study by (anonymized) and the Salud Mesoamerica Initiative?
2. Could you describe to me what you remember about the study (studies) in which you participated?

Section 1 Priorities and goals

1.1 How long have you been in this position?

1.2. What was your previous position?

1.3. Could you describe to me -in broad outline- the main activities you carry out in your current position?

1.4 What do you consider to be the priorities of the Ministry of Health in the primary health care system? What do you think of these priorities?

1.5. What are the priorities of your PHC team at this moment? How do they prioritize activities in the Unit to which you belong?

1.6. What is the role of the PHC teams in the health system of El Salvador?

1.7. Are you familiar with the Mesoamerican Health Initiative Program? If yes, is SMI currently supporting this Unit? Give me an example of how this support occurs? Based on your criteria, what has been the greatest contribution from SMI to this PHC team?

Section 2 - Performance Measurement

2.1. What is your perception of the work your PHC team is currently doing?

2.2. How do you know if your team is doing its job well or not?

2.3 The Salud Mesoamerica Initiative used to measure PHC team performance with some frequency. Rewards and recognition were often given to teams that were positively evaluated. Did you experience these?

Please describe those experiences.

How did you feel about those experiences (performance measurement and recognition)?

For peer review only

2.4. Are there other performance appraisal experiences?

2.5. How is your team's performance evaluated now?

Let's analyze the most recent example.

2.6. When did the appraisal occur?

2.7. Who did the evaluation?

2.8. How often is this done?

2.9. What data sources were used the last time one of these evaluations was conducted?

2.10. In the most recent evaluation of the team's performance, did the team receive any feedback?

2.11. How did you feel about this feedback?

2.12. How do you think the team felt? Elaborate on responses.

If feedback was provided:

2.13. How was the feedback provided? (Oral, written)

2.14. How often is feedback provided to your team about their performance?

2.15. How did you feel about this feedback?

2.16. What was done with the information contained in the feedback?

2.17. Were other team members made aware of the feedback? If yes, how was it shared?

2.18. Did the team do anything to correct their collective performance? Could you give me an example of an activity that was done?

If NO, comments or feedback were provided:

2.19. Would you like feedback on your team's performance?

Why?

2.20. Do you have any meetings with the MOH that you attend to discuss your team's performance?

If YES:

2.21. What is your opinion of these meetings, and why?

2.22. Are comparisons made between your team's performance and the performance of other teams?

Yes/no, please inquire:

2.23. How important would it be for you to attend these meetings? Do you feel it would be important to attend as a team? Why?

Section 3 - First Level of Care team performance management and individual work motivation

3.1. What aspects of your work as part of a PHC team are most rewarding to you?

For peer review only

3.1.1. Tell me about a recent work event that made you feel deeply rewarded or gratified.

3.1.2. Why did you feel this way? (Explore the 'why' in terms of emotions and feelings)

3.2. Sometimes the work is hard. Apart from COVID, what aspects of your work in a PHC team have been negative?

3.2.1. Tell me about a recent work event that affected you negatively.

3.2.2. Why did you feel this way? (Explore the 'why' in terms of emotions and feelings)

For peer review only

El Salvador decisionmakers at national and subnational levels

Introduction.

For the past decade, the Salud Mesoamerica Initiative has been working in collaboration with the Ministry of Health to promote maternal and child health in El Salvador. The overall objective of this study is to assess the ways in which MCH performance information is collected and used at the local and national levels in El Salvador. The team obtained non-objection from the Ministry of Health to conduct this research. The findings will be used to develop better health care practices in El Salvador and hopefully contribute to better health outcomes for Salvadorans.

Section 1 - Current Ministry of Health Primary Health Care Priorities

1.1. Tell me about the Ministry's priorities in the area of primary health care.

1.2. What are the strategic principles that guide the Ministry's PHC priorities?

Probe for:

1.2.1. Urban vs. rural; Comprehensive versus selective.

1.2.3. Efficiency and equity in the allocation.

1.2.4. Universalization versus targeting.

1.3. What role do the PHC teams now have in the functioning of the PHC system?

1.4. How well do you think PHC teams are doing their work?

1.5. What is the current role of SMI in relation to the priorities of the Ministry of Health in PHC?

Probe:

1.5.1. What is your perception of the value of MCH assistance to the MOH?

1.5.2. Could you give me an example?

Section 2 - Measuring PHC Team Performance

2.1 How does the Ministry evaluate the performance of the country's PHC system?

2.2 And in particular, how does the Ministry monitor the activities of the PHC teams?

2.3. Does the Ministry discuss any specific service delivery objectives with the teams?

2.3.1. What role do the teams play in the establishment of these objectives?

2.3.2. Describe the process (Investigate frequency, types of objectives, e.g., scope and/or facilities).

For peer review only

2.3.3. What performance indicators are now used?

Probe for:

- number of indicators used and request a list of key performance indicators in use.

2.3.4. Are performance targets used in all teams in the country? (If only the 75 teams in SMI's area of influence, ask why.)

2.4. In terms of service delivery, how does the Ministry evaluate the performance of the PHC teams?

Probe:

2.4.1. Using a recent example from a specific rural region, describe the process in detail.

2.4.2. What data sources did you use?

2.4.3. Who collects performance data from PHC teams?

2.4.4. Who analyzes the PHC team performance data?

2.4.5. How are the results presented to the PHC teams?

Section 3- Performance Management of PHC Teams

3.1. What are the PHC teams supposed to do with the results of the performance evaluation?

3.1.1. Does the ministry support the teams in understanding the results? How?

3.1.2. The Ministry has a long tradition of convening team managers and regional officers in frequent weekly or biweekly meetings to monitor progress. Is it still done? What are the results?

Section 4: Managing the overall performance of the PHC system

4.1 Beyond performance management of PHC teams, what else is the ministry doing with the results?

Probe:

4.1.1. Are the results used for planning? (If yes, ask for a recent example) 4.1.2.

4.1.2. Are the results used for budgeting? (If yes, ask for a recent example) 4.1.3.

4.1.3. Are the results used for any internal Ministry of Health reporting? (If yes, please ask for a recent example) 4.1.4.

4.1.4. Are the results used for MCH reporting? (If yes, please ask for a recent example) 4.2.

4.2. Does the MOH use aggregated data from PHC teams to adjust its priorities? How? Could you give me examples?

For peer review only

Lebanon interview guidelines - Health service providers and data collectors in the First Level of Care

Section 1- PHC Performance Measures

How is the aggregate performance of PHC system determined? Is there a performance assessment? What does it include?

Can you reflect on the processes and tools through which PHC system performance information is collected and processed? How would you characterize these?

Are there established performance indicators for PHC? If yes, can you share a sample of the performance indicators template (without any patient information or any other personally identifiable data)

How are the performance indicators selected? Which entity/department/unit/platform/team has input in this process (please provide only titles, not individual names)? Are these selected according to explicit criteria including usefulness, scientific soundness, reliability, representativeness, feasibility, accessibility?

Is there a guide/manual on how to calculate and interpret performance indicators? Does reporting on the performance indicators occurs on a regular basis?

How is information about performance measures communicated to you? For example, how did you first hear about them?

When a decision is made at the national level, how is it translated /monitored/implemented at subnational levels? Which entity/department/unit/platform/team is responsible for the communication from national to subnational level (please provide only titles, not individual names)? From subnational back to national level? How quickly does this happen?

What interventions or programs, if any, are implemented to ensure an enabling system environment for quality at level of PHC?

Registration and licensing; External evaluation/accreditation; Clinical governance; Public reporting and comparative benchmarking

For peer review only

Section 2- Performance Targets and Goals

2.1. How are performance targets for PHC set? Which entity/department/unit/platform/team is involved in the process (please provide only titles/department/unit, not individual names)? Are these typically set at a national, sub-national, or sub-regional level or left to the discretion of the facility?

Section 3- Data collection, analysis and sharing

3.1. Data collection

3.1.1. Is the data needed to construct the performance measure feasible to collect?

3.1.2. In what format is data collected? Is there a designated person to enter data/compile reports from the different units in the health facility?

3.1.3. What is the frequency of data collection? Is there a set data submission schedule?

3.1.4. Is there coordination of data collection across all PHC, e.g. via a unique identifier or a centralized data storage platform?

3.2. Data storage

3.2.1. In what database or file do you enter or store the data after you receive them? What computer program do you use? How frequently do you enter/store the data?

3.2.2. Any challenges in entering/storing the data? Any privacy issues when storing data?

3.3. Data aggregation

3.3.1 Do you perform any data aggregations? Which ones? Using which calculations? How often? Are there any challenges in aggregating data?

3.4. Data quality

3.4.1. What quality checks do you perform on the data? Is there a designated person to review the quality of compiled data prior to submission to the next level, e.g., to districts, to regional offices, to the central HMIS, etc.?

3.4.2. Are there any known issues with data quality?

3.5. Data reporting & sharing

3.5.1. Are there written guidelines available on reporting protocols, including the following: what they are supposed to report on; how reports are to be submitted, e.g., in what specific format; to whom the reports should be submitted and when the reports are due?

3.5.2. In what format do you share the data (type of form, file, database, aggregation levels)? Are data visuals prepared (graphs, tables, maps, etc., balanced scorecards) showing achievements toward targets (indicators, geographic and/or temporal trends, and situation data)?

3.5.3. With whom do you share the data? How often do you share the data?

For peer review only

3.5.4. How do you share the data (e.g., internet, email, USB flash drive, disk, hard copy)?

3.5.5. Are there any delays or challenges in preparing data & sending them?

Section 4- Performance Monitoring & Feedback

How is progress towards the performance targets monitored/tracked? Can you give us an example? Is this considered routine?

Is there a performance monitoring or management team?

Which entity/department/unit/platform/team is the most responsible for managing or tracking progress for performance measures? (please provide only titles, not individual names)

Which entity/department/unit/platform/team do they report to/who manages this process? (Please provide only titles, not individual names)

Which entity/department/unit/platform/team is in charge of the data pulls and reports? (please provide only titles, not individual names)

Which entity/department/unit/platform/team do the data analysts and the performance manager report to? (Please provide only titles, not individual names)

Section 5- HIS and data quality assessment

5.1. What specific information flows has the organization in place to support the performance management systems? Are systems interoperable so that information can be shared across geographic areas, types of facilities, etc.?

5.2. Are staff trained in how to use the information systems (not only collecting data, but also collating, analysing and interpreting data)

5.2.1. Do these staff typically have dedicated time allocated for using information systems and interpreting data?

5.2.2. Do they have capacity to explain the implications of the results of data analysis?

5.2.3. Are the information systems accessible to these individuals?

5.3. Are there data quality assessment mechanisms in place?

5.3.1. Are there written instructions/guidelines on how to perform a data quality review or data quality check?

5.3.2. Do data management staff conduct regular checks of the accuracy and completeness of data prior to submitting reports to the next level (using automated electronic checks, where appropriate)?

5.3.3. Does the health facility receive periodic feedback from higher levels (e.g., MOPH) on data quality?

For peer review only

5.3.4. What is your perception of the quality of existing data on PHC performance? How can it be improved?

Section 6- Supervision & Feedback on performance

Do you receive technical support or supervision in your work?

Which entity/department/unit/platform/team conducts supervision? What sort of support of qualifications do these individuals have? (please provide only titles, not individual names)

Which entity/department/unit/platform/team receives supervision? Which ones do not? (please provide only titles, not individual names)

Where do supervisory visits take place and how long do supervisory visits last?

What sort of activities do supervisory visits cover? Does the supervisor use a standard checklist for assessment?

How frequently does supervision occur?

Is there an established schedule for supervision, or do visits need to be requested?

Does the supervisor send report/ written feedback on the past supervisory visit(s)?

Do you get feedback on your performance? How? Frequency? What does it look like? How would you improve this process?

Feedback on data quality (including data accuracy, reporting timeliness, and/or report completeness)

Feedback on service performance based on reported data (e.g., appreciation/acknowledgement of good performance; resource allocation/mobilization)

What, if any, feedback loops exist between the facility level, regional, sub-national level and national level and how do they function?

What rewards — financial and/or non-financial — will managers and other employees gain by achieving performance targets or other assessed aspects of performance (and what penalties will they suffer by failing to achieve them)?'

Section 7- Implementation of quality improvement activities

Are discussions often held to review key performance targets (tracking progress against targets) based on performance measure information?

Are quality improvement activities implemented to take corrective action?

How are the objectives of quality improvement activities set?

For peer review only

Which entity/department/unit/platform/team initiates these activities? (please provide only titles, not individual names)

Which entity/department/unit/platform/team monitors them? (please provide only titles, not individual names)

Section 8- Overall perceptions on collection of performance measure information

8.1. To what extent to do you agree with the following:

I can use data for identifying performance gaps and setting targets

I feel discouraged when the data that I collect/record are not used for taking action (either for monitoring or decision making)

I find collecting/recording data to be tedious (i.e., repetitive or duplicative)

I find that the data that I collect burdens my workload, making it difficult for me to complete my other duties

Collecting data is meaningful/useful for me

I feel that the data I collect are important for monitoring the performance of the health services provided at my facility/unit

My work of collecting data is appreciated and valued by decision-makers at national and subnational level

For peer review only

Lebanon decisionmakers at national and subnational levels

Section 1- PHC Performance Measures

How is the aggregate performance of PHC systems measured (organizational and whole-system level)?

How do you track progress toward goals? Can you show us an example?

Which entity/department/unit/platform/team are most responsible for managing or tracking progress for performance measures? (please provide only titles, not individual names)

Which entity/department/unit/platform/team do they report to/who manages this process? (please provide only titles, not individual names)

Which entity/department/unit/platform/team is in charge of the data pulls and reports? (please provide only titles, not individual names)

Which entity/department/unit/platform/team do the data analysts and the performance manager report to? (please provide only titles, not individual names)

What rewards — financial and/or non-financial — will managers and other employees gain by achieving performance targets or other assessed aspects of performance (and what penalties will they suffer by failing to achieve them)?

Section 2- Awareness of data flow at different levels

2.1. Are you aware of how PHC performance measurement information is collected and processed at different levels?

2.2. Can you reflect on data flow from facility level to district/subnational to MoPH? What is your perception of the process?

Section 3- Data format

3.1. In what format do you receive performance measure information?

3.1.1. Are data visuals (e.g. summary tables, graphs, geographic information system, maps, pivot tables, decision support systems, etc.) showing achievements toward targets (indicators, geographic and/or temporal trends, and situation data) prepared and used to display information at MOPH level and within health facilities. They are up to date and clearly understood

3.1.2. Are reports or bulletins (annual, quarterly, etc.) periodically produced based on an analysis of performance measurement information and distributed to key stakeholders? Do they contain discussions and decisions/recommendations based on key performance targets?

For peer review only

3.1.3. Are there challenges with interpreting the information?

3.2.3. What is the frequency of receiving performance measure information? Is there a set schedule for receiving such information? What is your perception of the process?

3.2. How do you receive the performance measure information (e.g., internet, email, USB flash drive, disk, hard copy)? In what database or file is data stored or entered after you receive them?

3.3. What quality checks do you perform on the data? Are there any known issues with data quality?

3.4. How could (or should) health information products look like for policy-makers? and at what level of disaggregation and/or in which quality?

3.5. With whom do you share the data? In what format do you share the data (type of form, file, database, aggregation levels)? How do you share the data (e.g., internet, email, USB flash drive, disk, hard copy)?

Section 4- Feedback on data received

4.1. When a decision is made at the national level, how is it translated /monitored/implemented at subnational levels? Which entity/department/unit/platform/team is responsible for the communication from national to subnational level (please provide only titles, not individual names)? From subnational back to national level? How quickly does this happen?

4.2. Is feedback systematically provided to all sub-reporting units on the quality of their reporting (that is, accuracy, completeness, and timeliness)?

4.3. Is feedback systematically provided to all sub-reporting units on the use of performance measure information for decision-making?

Section 5- Decision-making process

5.1. How are decisions made at your institution? What factors have strong influence on decision-making process:

Personal preference of decision makers

Superiors' directives

Evidence/facts/data (RHIS data)

Funding directives from higher levels

Political considerations

Official health sector strategic objectives

Locally identified health needs of the population

The relative cost of interventions

For peer review only

5.2. Is there a mandate to use data to inform decision-making? What incentive structures are in place to promote the use of data in decision-making? Are there any consequences for NOT using data to inform critical decision-making?

Section 6- Use of data in decision-making process

6.1. Can you reflect on the processes through which decision makers (at national and district levels) make sense of, and use PHC system performance information? What is your perception of the process?

6.2. Do the existing performance measure information respond to policymakers' priorities and needs? Are data producers and users brought together periodically to discuss ways of making routine data more relevant to policy makers and planners and to enhance the understanding of routine health statistical findings?

6.3. How is aggregate PHC system performance information used by decision makers at the national and district level?

6.4. Were any decisions made based on the discussions of the performance measurement information? Such as:

Advocacy for policy, programmatic, or strategic decisions

Formulation of plans

Budget preparation/reallocation

Medicine supply and drug management

Human resource management (training, reallocation, etc.)

Promotion of service quality/improvement, equity

Section 7- Impact

7.1. Would your organizational priorities be different without performance measures? How? How do measures affect your relationship or interactions with staff?

7.2. How have performance measures changed the way you operate?

7.3. How do performance measures affect policy decisions? Budget decisions? Staff decisions? Scheduling? Education/training/orientation?

7.4. To what extent is (staff) compensation linked to performance measures? Can you explain how that works? How does this affect your perception of performance measures?

For peer review only

Section 8- Perceptions on data use culture

8.1. What is your perception of the prevailing culture for data use at your institution?

Probes:

A culture of information use is promoted by policy leaders and decision-makers, and is reflected in the use of facility and community-based data in planning, monitoring, and evaluation reports

Performance measure information is readily available in written periodic reports or bulletins that pulls together and analyses critical health information from all subsystems

Senior managers and policymakers demand complete, timely, accurate, relevant and validated HIS information

Policy and decision makers regularly use performance measure information to evaluate performance and set policies on health.

Data visualizations are widely used to display information at subnational health administrative offices and health facilities. They are up to date and clearly understood

Performance measurement information is demonstrably used in the national planning and in the resource-allocation processes (e.g., for annual integrated development plans, medium-term expenditure frameworks, long-term strategic plans, and annual health sector reviews)

Performance measure information is widely used, by sub-national management teams to set resource allocation in the annual budget processes

Performance measure information is used to advocate for equity and increased resources to disadvantaged groups and communities

Managers at all levels use health information for health service delivery management, continuous monitoring, and periodic evaluation

Section 9- Big Picture

9.1. How would you characterize the use of performance health information in Lebanon? All in all, do you think there is too much/not enough emphasis on performance measures?

9.2. What are the challenges that hinder the use of performance measurement information to inform health policymaking and decision-making processes?

9.3. Do you have suggestions on how to improve the role of performance measure information in informing the policymaking process?

Relevance

Quality

Timeliness/Accessibility

Analysis and interpretation

For peer review only

Format

9.4. Can you suggest up to three policies/ strategies/mechanisms that can help improve your institution’s capacity to use data in policy and program development and implementation?

For peer review only

Malawi – Interview guide for Decision Makers

Participant details

Facilitator's Name: ______________________Date of the interview ______________

Name of the Facility:___________________ Location ______________

Respondent's age____________________ Respondent's Sex_________

Respondent's Position _________________ Qualification______________

Respondent's years of experience____________________________

For peer review only

Introduction

What is your role in this organization?

What is your qualification

How long have you worked in this organization?

How long have you worked at your current position?

What are the main priorities of MOH in PHC?

What are your PHC priorities?

Currently, how can you rate your impact in PHC? Is your performance improving or not? How do you know?

Knowledge about data collection and use

Do you know how PHC data is collected at your institution?

If yes, may you briefly explain how it is done?

Do you think PHC data is collected in honest and effective manner?

Why is data collection and use important in your organization?

How is data utilized in policy formulation?

Performance Measurement

Please describe the ways in which the performance of PHC at your level is evaluated? Specifically, let us look at

Who does the evaluation?

When did the evaluation happen?

What data was used to assess the team's performance

How frequently is this done

When the performance assessment is complete, what type of feedback do you receive (oral or written)

How frequently do you receive the feedback about your performance?

What do you do with the feedback you receive about the PHC performance? May you give an example of a recent situation in which you used the performance results in your daily practice and services

Probe on: a. planning b. budgeting c. policy formulation

Do you have any specific example of a situation in which PHC performance results did not help you?

What do you think are the challenges associated with performance measurement?

What do you think can be done to address the challenges?

Factors affecting data driven decision making (DDDM)

May you briefly explain the things you consider when making a decision in your organization?

Probe on: data consultation, political factors, group/personal influence etc

Why are these things important in decision making?

Have you ever made important decision without relying on the available data?

If yes, why was data not relevant for that decision?

If not, why is relying on data important?

Are there factors/reasons for making a decision without relying on data?

Probe on: lack of data authenticity/trustworthiness, political influence, lack of understanding of data, lack of time to study the data, negligence etc

What do you think are the challenges associated with data driven decision making?

What do you think can be done to address the challenges?

Theme 3: Policies on data driven decision making

Are there policies that guide managers on data driven decision making?

If not, what drives managers to make a decision by relying on data?

Are all policies in PHC made in consultation with data?

If not, why?

What could be the result of formulating a policy without consulting data?

What does the ministry do?

Challenges and recommendations

Do you have any challenges on data collection and use in your organizations?

For peer review only

If yes, what are the challenges?

Probe on: lack of understanding of the data, bulkiness of the data, lack of time to study the data, lack of data authenticity, political interference etc

What do you think should be done to improve data collection and use in your organization?

Probe on: a. what should the data collectors and users do?

b. what should the government do?

Thank you very much. This is the end of our discussions.

For peer review only

BMJ Open

Characterising performance information use in the primary health care systems of El Salvador, Lebanon, and Malawi: Multiple qualitative case study protocol

Journal:	*BMJ Open*
Manuscript ID	bmjopen-2021-060503.R1
Article Type:	Protocol
Date Submitted by the Author:	13-Sep-2022
Complete List of Authors:	Munar, Wolfgang; George Washington University, Global Health<br>Wahid, Syed; George Washington University, Division of Global Mental Health<br>Makwero, Martha; University of Malawi College of Medicine, school mof public health and Family medicine; Queen Elizabeth Central Hospital, Obstetrics and Gynaecology<br>El-Jardali, Fadi; Department of Health Management and Policy, Faculty of Health Sciences, American University of Beirut, Beirut, Lebanon, and 2Sch,<br>Dullie, Luckson; Partners In Health,<br>Yang, Wen-Chien; The George Washington University Milken Institute of Public Health, Global Health
**Primary Subject Heading**:	Global health
Secondary Subject Heading:	Health policy
Keywords:	Organisation of health services < HEALTH SERVICES ADMINISTRATION & MANAGEMENT, Health policy < HEALTH SERVICES ADMINISTRATION & MANAGEMENT, PRIMARY CARE

SCHOLARONE™ Manuscripts

Characterising performance information use in the primary health care systems of El Salvador, Lebanon, and Malawi: Multiple qualitative case study protocol

Author list:

Wolfgang Munar, George Washington University, Milken Institute School of Public Health, Washington, DC, United States of America

Syed Shabab Wahid, Department of Global Health, Georgetown University, Washington, DC, United States of America

Martha Makwero, Kamuzu University of Health Sciences, School of Medicine and Dentistry, Blantyre, Malawi

Fadi El-Jardali, American University of Beirut Faculty of Health Sciences, Beirut, Lebanon

Luckson Dullie, Partners in Health, Malawi

Wen-Chien Yang, George Washington University, Milken Institute School of Public Health, Washington, DC, United States of America

Corresponding author:

Wolfgang Munar
Milken Institute School of Public Health
950 New Hampshire Avenue NW
Washington DC 20552, United States of America

Email: wolfgangmunar@gwu.edu

Phone: +1.206.218.7298 (Mobile) +1.202.994.1015 (Office)

Keywords

Performance evaluation; Performance measurement and management; Performance information; Organisation and management of care; Decision making

Wordcount: 3,946, including abstract

For peer review only - http://bmjopen.bmj.com/site/about/guidelines.xhtml

ABSTRACT

Introduction. Governments in low- and middle-income countries (LMICs) and official development assistance agencies use a variety of performance measurement and management (PMM) approaches to improve the performance of healthcare systems. The effectiveness of such approaches is contingent on the extent to which managers and care providers use performance information. To date, major knowledge gaps exist about the contextual factors that contribute, or not, to performance information use by primary healthcare (PHC) decisionmakers in LMICs. This study will address three research questions: 1) How do decision makers use performance information, and for what purposes? 2) What are the contextual factors that influence the use or non-use of performance information? and 3) What are the proximal outcomes reported by PHC decisionmakers from performance information use?

Methods and analysis. We present the protocol of a theory-driven, qualitative study with a multiple case study design to be conducted in El Salvador, Lebanon, and Malawi. The study entails semi structured in-depth interviews and document review. Interviews will be conducted with approximately sixty respondents including PHC system decisionmakers and providers. We follow an interdisciplinary theoretical framework that draws on health policy and systems research, public administration, organisational science, and health service research. Data will be analysed using thematic analysis to explore how respondents use performance information or not, and for what purposes as well as barriers and facilitators of use.

Ethics and dissemination. The ethical boards of the participating universities approved the protocol presented here. Study results will be disseminated through peer-reviewed journals and global health conferences.

For peer review only

For peer review only - http://bmjopen.bmj.com/site/about/guidelines.xhtml

Strengths and limitations

- Strengths include the use of theory to guide study design, data collection, and reporting; the consideration of rival explanations; and the use of triangulation of data sources, respondent accounts, and researcher interpretation.
- The use of a case study design with embedded units in different country contexts can contribute to theoretical generalizations about the influence of contextual factors on performance information use and non-use, and thus influence future comparative research.
- Limitations include reduced transferability of findings to contexts other than the three participating countries and to other populations of decision makers and providers. The use of virtual interviews may create potential loss of rapport between interviewers and respondents. The use of virtual interviews may create potential loss of rapport between interviewers and respondents.

w only

For peer review only - http://bmjopen.bmj.com/site/about/guidelines.xhtml

I. Introduction

This protocol aims to describe how decision makers and providers in three low- and middle-income countries use available data to assess the performance of their PHC systems. Acquiring this knowledge is important for improving PHC systems responsiveness and can contribute to the achievement of Universal Health Coverage in the era of Sustainable Development Goals. High-performing PHC systems have also proven to be key in the preparedness for and response to pandemics and other public health emergencies (1, 2).

PHC has been defined as a whole-of-government and whole-of-society approach that combines multisectoral policy and action, empowered people and communities, and primary care and essential public health functions as the core of integrated health services (3). PHC systems are first points of entry into health service delivery, are essential for people-centred service delivery, and connect citizens to health systems (4).

During the last forty years, PMM systems have become prevalent in healthcare management and organisation (5-7). Governments, official development assistance agencies, and various global health partnerships have used diverse PMM approaches to improve performance of policies and programs in maternal and child health (8, 9), HIV/AIDS, malaria, and tuberculosis (10), and other global health priorities. Outcomes-driven financing approaches have also been used as a means to improving PHC system performance (11).

Performance measurement and management systems

PMM systems were originally conceived as ensembles of management control mechanisms designed to stimulate the delivery of organisational priorities and influencing desirable organisational behaviours (12-14). However, depending on contextual factors and historical antecedents, PMM systems have evolved in response to contrasting organisational logics (15). *Directive* systems tend to be guided by a logic of consequences, are prevalent in systems that

For peer review only - http://bmjopen.bmj.com/site/about/guidelines.xhtml

favour audit cultures (16), are designed with a view towards accountability, and follow the utility-maximising assumptions of *Homo economicus* in agency theory (17). *Enabling* approaches, on the other hand, are guided by logics of improvement and learning; can create conditions for adaptive and iterative cycles of error, reflection, sensemaking, and corrective action; and conceive of performance as emergent processes, influenced by managers and workers' agency, motives, means, and opportunities (18, 19).

Studies on PMM systems' effectiveness have identified several sources of leverage for performance improvement in public sector organisations (20-23). Organisational performance tends to be positively associated with PMM systems that reinforce workforce motivation (24); promote performance measurement at multiple levels (i.e., individual, interpersonal, and inter-organisational) (25); and where decisionmakers use of the information generated through performance appraisals (26, 27).

Governments use and official development assistance agencies promote a diverse set of approaches to performance management including multifaceted financial arrangements, accountability approaches, and implementation strategies (28). An evidence gap map of PMM interventions in the PHC systems of LMICs showed that most primary studies to date have focused on provider-level implementation strategies such as in-service training and supervision, and on financial arrangements like pay-for-performance (29). The mapping exercise also identified absolute gaps in evidence for PMM interventions that operate at organisational levels, particularly accountability arrangements like public release of performance information or social accountability. There is also limited knowledge about the role of contextual factors in enabling or hindering the use of performance information at the organisational level of teams, facilities, and district health systems. Table 1 summarises the interventions mapped in the evidence gap map above.

For peer review only

For peer review only - http://bmjopen.bmj.com/site/about/guidelines.xhtml

The widespread use of PMM systems in the public sector, particularly in health, has shown that, when not tailored to context, PMM systems can not only be ineffective but can also contribute to negative outcomes such as gaming, goal displacement, and data manipulation (30). Further, public administration research has also shown that decision makers do not consistently use performance information and that, when they do, the largest impacts on service delivery are attained when it is used as part of organisational dialogues that inform changes in operational and strategic direction (26, 27, 31). The literature has also shown that official development assistance agencies promote and use various PMM approaches for improving accountability to donors and beneficiaries; enhancing organisational learning and communications; and informing changes in strategic direction (32).

Table 1. Interventions and approaches in PHC systems performance measurement and management

Implementation strategies	Accountability arrangements	Financial arrangements
Provider-level: Clinical practice guidelines, reminders, in-service training, and continuous education<br>Organisational level: Clinical incident reporting; clinical practice guidelines; local opinion leaders; continuous quality improvement; and supervision	Individual- or organisational-level: Audit and feedback<br>Community-level: Public release of performance information, social accountability	Individual and organisational level: Results-Based Financing, Pay for Performance, and other provider incentives and rewards.

The literature on routine health information systems (RHIS) in LMICs has identified organizational, behavioural, and technical challenges to the production and use of information including, among others, fragmentation, duplication, and poor data quality (33). It has also been shown that even when quality health information is available, LMIC health managers may not use it, leading to suboptimal decision-making processes that may negatively affect governance and healthcare management. Previous research has also found that non-use of data from RHIS

For peer review only - http://bmjopen.bmj.com/site/about/guidelines.xhtml

can be explained by lack of motivation or scarce capacity among decision makers; and by non-existing or poorly functioning feedback and supervision mechanisms (34-37).

To address these gaps in literature and increase the understanding of performance information use, or not, in LMIC settings, this article presents the protocol of a multisite, qualitative case study.

II. Methods and analysis

For peer review only

Study aims and research questions

The study described here will assess the experiences of PHC decisionmakers and providers with performance measurement and management in El Salvador, Lebanon, and Malawi. Research findings will be used to inform an applied research agenda on PHC system performance; contribute to improve the measurement and management of PHC systems performance; and develop an evaluation framework for assessing performance information use in other country contexts. Our research questions are: 1) How do PHC system decision makers use performance information, or not, and for what purposes? 2) What are the contextual factors that influence the decision to use performance information, or not? 3) What are the proximal outcomes reported by PHC decision makers from performance information use and non-use?

Theoretical framework

Based on PMM models in public administration research, implementation research, and organisational science (6, 22, 38-40) we developed an interdisciplinary theoretical framework to help guide study design. PMM systems are conceptualised as continuous and recursive cycles of 1) organisational priorities and goals; 2) incentives; 3) performance measurement, feedback,

For peer review only - http://bmjopen.bmj.com/site/about/guidelines.xhtml

and sense-making; 4) implementation strategies; and 5) performance outcomes (6), as represented in figure 1.

Figure 1

Organisational priorities and goals are the ultimate expression of what desirable performance ought to be; they are identifiable in policy documents, summarized in logical models, and sometimes reflected as measurable targets in performance frameworks. Incentive systems are managerial practices aimed at stimulating workforce motivation and fostering organisational performance by means of extrinsic and intrinsic stimuli. Extrinsic motivators include rewards, recognition, pay for performance, bonuses, and in-kind incentives, among others (41). Intrinsic motivators aim to trigger satisfaction of workers' basic needs such as competence, autonomy, and connection (42, 43). It is believed that both types of motivators are central to organisational performance (44).

Performance measurement processes generate raw data about past performance and use metrics that reflect organisational priorities and goals. Performance data are usually compiled into registers that feed into routine health information systems (RHIS), and can be summarised and disseminated via reports, scorecards, and dashboards. Given the perceived low-quality of RHIS, particularly in LMICs (35), performance data is also sourced from cross-sectional population surveys. The latter have become one of the most frequently used data sources for tracking health programs' performance (36, 37).

The data acquired via RHIS and/or population surveys are usually contrasted against expected targets and goals which, in turn, are disseminated in ways that generate performance information flows aimed at different users. Upward flows bring information through organisational hierarchies usually for reporting and accountability purposes. Information can also be fed-back to the frontlines of service provision as part of feedback and audit, quality improvement, or supportive supervision processes (45). As organisational actors engage with

For peer review only - http://bmjopen.bmj.com/site/about/guidelines.xhtml

performance data, ascribe meaning to it, and imagine future courses of action in response to perceived gaps in performance, the managerial processes above can contribute to collective sensemaking (46), a process that helps people 'understand issues or events that are novel, ambiguous, confusing or in some other way violate expectations' (47). It can also inform decisions among organisational actors to engage or not in addressing the gaps in performance made evident by available information. Action plans, budgets, changes in service delivery, and other processes of course-correction can thus be considered for future implementation.

Once courses of action are decided, organisational actors can deploy various strategies to implement them. Implementation strategies help system actors appraise and respond in adaptive fashion to factors in their immediate environment that can enable or hinder collective action (see table 1). In the short term, performance information can be used for planning, compliance, reporting, or rapid course-correction purposes, among others; it can also be misused through gaming processes, or not used (30). As iterative PMM cycles are repeated through time, performance information can also be used (or not) as the basis for testing new processes and services, for internal advocacy, and/or for policy formation.

PMM cycles can contribute to proximal performance outcomes that feed into long causal chains of outcomes occurring at multiple levels within an organisation (e.g., at individual, team, and organisational levels). Outcomes can include 1) proximal changes resulting from using performance information (or not), such as action plans implemented, compliance with procedural standards, timely reporting, and rapid course-correction; 2) intermediate effects emerge at the organisational behaviour level, and may include changes in workforce motivation, job satisfaction, morale, or organisational commitment; and 3) downstream population-level health effects and equity outcomes resulting from the iterative repetition of PMM cycles in dynamic and changing environments.

For peer review only - http://bmjopen.bmj.com/site/about/guidelines.xhtml

We integrated the elements of the PMM model described above into a theoretical framework that represents the hypothetical process of performance change and the role played by performance information use and non-use. The framework contains the following elements: external context; PMM approaches in use within the public sector; performance information production and use; PHC systems' internal organisational environment; and the causal pathways connecting performance information use and non-use to proximal, intermediate, and distal outcomes.

The theoretical framework is represented in figure 2. Here, the managerial practices used to measure and change performance are influenced by external and internal contextual factors and by the implementation strategies in use and modulated by the use and non-use of performance information. The processes of change thus generated can contribute, via long causal chains, to a variety of outcomes and impacts. Proximal effects from performance information use are represented by single-loop learning effects (48) such as changes in planned action, rapid course-correction, and improvements in service quality. The repetition of such iterative cycles may, in turn, contribute to the emergence of second-loop learning effects such as changes in strategic direction and new practices among service providers and managers (49).

Figure 2

The use of performance information is causally linked to proximal performance outcomes at the individual level of providers and patients; those outcomes are also causally connected to intermediate outcomes at the organisational level such as improved workforce motivation, enhanced organizational commitment, increased trust between providers and PHC system users, and reduced staff turnover, among others. These outcomes can contribute to distal population health and equity outcomes (intended and otherwise). Depending on context, the causal chain of outcomes described above can also be interrupted, be limited to isolated pockets of excellence, or be altogether absent.

For peer review only - http://bmjopen.bmj.com/site/about/guidelines.xhtml

Study design

The present study will explore the uses of performance information in the PHC systems in El Salvador, Lebanon, and Malawi. Investigation across different contexts allows for the generation of context-specific insights of value to local actors and, potentially, to broader understandings of the phenomena of interest (50).

To address the research questions, we chose a theory informed, multiple case study design with embedded units of analysis (51). Case studies are well-suited for obtaining an in-depth understanding of context-specific processes in complex systems (52). Here, a case is defined as each country's PMM practices; the two units of analysis included are PHC service provision and PHC policy implementation at national and subnational levels.

Study setting

El Salvador

El Salvador is a lower-middle income country with a population of 6.4 million. Since the conclusion in 1992 of a civil war, the country reduced inequality by about 5 percentage points between 2007 and 2016; increased coverage of institutional deliveries and immunisation to 98% and 93%, respectively; and achieved the under-five mortality reduction for the Millennium Development Goals (53, 54).

Starting in 2009, El Salvador universalised access to free, comprehensive PHC. Existing infrastructure was reorganised into PHC networks, one for each of the Departments in which the country is administratively divided. Service delivery was delegated to multi- professional teams of PHC providers. The oversight of each Departments' network is the responsibility of a decentralised MOH coordination team called SEBASI in its Spanish acronym. PHC teams have a nominal catchment area of 3,000 individuals and are co-located within the communities they

For peer review only - http://bmjopen.bmj.com/site/about/guidelines.xhtml

serve. A basic PHC team is made up of one medical doctor, two nurses, and up to three community health promoters; some teams have specialised care providers. PHC teams provide community outreach as well as facility-based services and deliver a package of benefits containing approximately 300 interventions (55).

In 2011, the government of El Salvador joined the Salud Mesoamerica Initiative (SMI), a public-private partnership focused on improving the performance of PHC systems in the eight nation states of Mesoamerica. In El Salvador, SMI operates in 75 PHC teams operating in the poorest rural municipalities in the country. PMM interventions used include PHC team target-setting; monitoring of PHC teams' performance using population and facility surveys and RHIS; provision of feedback to teams; and team-based in-kind incentives (55, 56).

Lebanon

Lebanon is home to approximately 6,8 million people and is classified as an upper-middle income country (57). However, the financial crisis that started in 2019 reduced real per-capita gross domestic product 37.1 percent between 2018 and 2021 The country also hosts the largest number of refugees per capita in the world (58) and has suffered additional internal shocks. The combined effect of these various shocks has put major pressure on an already stretched healthcare system (59, 60).

PHC services in Lebanon are provided by a combination of private-for-profit and not-for-profit providers; the latter are the most accessible and used sources of care by vulnerable Lebanese and refugee populations (61, 62). Lebanon's official PHC network is comprised of 213 centres that have contractual agreements with the Ministry of Public Health based on pre-met community care delivery standards.

In terms of performance measurement at the PHC level, the MOH has developed policies and practices to monitor service delivery patterns, quality of care and performance of PHC centres

For peer review only - http://bmjopen.bmj.com/site/about/guidelines.xhtml

within the national network (63). Monitoring involves regular visits by MOH inspectors and administration of patient satisfaction surveys (63). Accreditation is also used to regulate the quality of care at the PHC level. By establishing a National Accreditation Program for PHC centres in 2009, the MOH aimed to ensure continuous and sustainable quality control, improve compliance with legal and safety standards, enhance transparency and accountability, and establish a positive image of standards of practice and service at PHC centres (63).

Despite the various health reforms implemented in Lebanon, there is still no active national strategic plan designed around PHC (64-67). Furthermore, many PHC centres remain underdeveloped with no availability of basic diagnostic imaging and laboratory medicine, resulting in perceived lack of confidence on the quality of services offered (60).

Malawi

Malawi is a land-locked, low-income country with a population of approximately 18.6 million. The economy is mainly dependent on the agricultural sector which employs 80% of the population. A five-year development plan, Malawi's Growth and Development Strategy, guides the country's development; the current plan is focused on education, health, agriculture, energy, and tourism (68).

Malawi's epidemiological profile combines high burden of disease from both preventable conditions as well as noncommunicable diseases. The country has a high population density and a total fertility rate of 4.4. Prevalent social determinants of health include poverty and inequality, high levels of illiteracy and limited coverage of social safety programs (68, 69).

Primary care is the main platform for the delivery of health services in Malawi. However, the PHC system is characterised by poor distribution of human and physical resources, fragmentation of services and chronic shortages of staff (70). To reduce service fragmentation, Malawi developed in 2017 a new community health policy centred on a team-based approach.

For peer review only - http://bmjopen.bmj.com/site/about/guidelines.xhtml

For peer review only

Community health teams (CHT) comprise health surveillance assistants (HSAs), clinicians, environmental health officers and community health volunteers (71).

Data collection

The proposed study will utilize document review and semi-structured interviews with informants who typically hold 'great knowledge…[and] who can shed light on the inquiry issues' (72). We will use document review to identify domestic priorities and explore the external context, available resources, and ongoing official development assistance programs. Documents to be reviewed include MOH policy documents, strategic frameworks, operational plans, results frameworks, performance reports, and logical models, among others. Semi-structured interviews will be conducted with PHC decisionmakers at the national and subnational levels and with PHC providers. To be eligible for inclusion, decisionmakers will be current or former officials responsible for PHC system policy formulation or implementation at national and subnational levels; providers will be staff currently working as clinical care providers or community health workers.

Respondent selection and recruitment will follow an information power approach based on criteria that are suitable for reaching saturation in qualitative studies using non-probabilistic, purposive sampling (73). We will design respondent sampling guided by our understanding of the types of participants that can provide highly specific information to address the study's research questions; insights from the preliminary theoretical framework; and responsive to the quality of the dialogue elicited during data collection. The estimated number of respondents to be interviews in the three countries is approximately 20 respondents per site, for a total of 60 respondents. However, sampling numbers will be further refined, and may be expanded, based on preliminary analysis of data as data collection is ongoing. Respondent inclusion criteria will

For peer review only - http://bmjopen.bmj.com/site/about/guidelines.xhtml

be calibrated to the context of each study setting; site-specific approaches to data collection will be reported in each country case study.

In the interviews with service providers, we will explore experiences about the PHC system organisational environment; the ways in which PHC performance is measured, analysed, and made sense of; the extent to which performance information is used or not, and for what purposes, and the reported effects from using performance information. Interviews with decisionmakers at national and subnational levels will explore PHC priorities, goals and/or targets; characterise the public sector institutional context; explore sources and frequency of performance data appraisal; and inquire about the uses of performance information. We will also triangulate the data resulting from document review and interviews, and the experiences reported PHC by the two types of respondents.

In each country, the research team will develop a Project Brief summarizing the study's aims and highlighting the voluntary nature of participation. An invitation to participate in the interview will be sent individually via e-mail to each potential respondent. Once the respondent agrees to participate in the interview, a remote interview will be scheduled (or in-person, if allowed by ethical review board). Before initiation of the interview, the interviewer shall read the consent form and obtain verbal consent from the interviewee which will be recorded and reflected in the interview transcript accordingly. Site-specific interview guidelines are available in supplementary file 1.

Analysis

Interviews will be audio taped, transcribed verbatim and imported into NVivo 12.0. Transcripts will be coded independently by at least two researchers in each country. We will use an iterative, directed approach to analysis (74) informed by the theoretical framework. The latter shall also inform the design of a codebook to guide deductive coding of the data. Inductive

For peer review only - http://bmjopen.bmj.com/site/about/guidelines.xhtml

codes emerging from the data will also be identified and included in the analysis. We will convene analytic workshops among the research teams in participating countries to discuss the codebook, the coding process, thematic analysis, and data synthesis procedures.

After the conclusion of coding in each country, we will execute code queries for each code, stratified by respondent type (e.g., providers and decision makers) to extract code-specific data. Subsequently, we will review and summarize the code-specific and respondent-specific data from the query outputs into code summary memos using a standardized template.

Code summary memos will include a respondents table to capture brief and relevant information from each type of respondent, and narratives constructed by the researcher reviewing the query output, supported by exemplary quotes. Code summary memos will include deviant narratives and quotes that run counter to the main narrative(s) and a section for recording researcher insights on where and how codes may be connected to others. In a final step, the synthesized data in the code summary memos will be organized into thematic matrices to formalize linkages between codes and construct themes. The resulting themes will be used to report country-specific findings and to develop a refined theoretical framework. Results for each country case will be organised using the SRQR checklist (75) (Supplementary file 2). To increase credibility in our findings we will consider rival explanations and triangulate across data sources (i.e., SMI relevant program documents and in-depth interviews), respondents (decision makers and providers), researchers, and social and behavioural science theories. Data collection and analysis will take place between June 2020 and June 2022.

The proposed study has several strengths including the use of theory to guide study design, data collection, and reporting; the consideration of rival explanations (76); and the use of triangulation of data sources, respondent accounts, and researcher interpretation (77). Case study research has limitations including reduced transferability of findings to other contexts and

For peer review only - http://bmjopen.bmj.com/site/about/guidelines.xhtml

different populations of decision makers and PHC providers (76). Also, the use of virtual interviews may create potential loss of rapport between interviewers and respondents.

Patient and public involvement

Neither patients nor public were involved in the conduct, reporting or dissemination of the research presented in this protocol.

III. Ethical considerations & dissemination

The ethical approval for this study was provided by the Institutional Review Boards of the participating universities (Study numbers NCR203102 for the George Washington University; SBS-2021-0162 for American University in Beirut, and P.11/20/3198 for the University of Malawi). We will follow ethical principles of voluntary and informed involvement in the study, confidentiality, and safety of all participants. Verbal consent will be obtained from all respondents and be reflected in the respective interview transcripts.

A database will be maintained containing information on all interviews completed, including demographic data and time of the interview as well as confirming verbal consent by each respondent. All identifying information will be stored in an encrypted database, hosted in encrypted and password protected cloud services provided by each of the hosting research institutions. The identifier information database will be permanently deleted after the completion of data analysis.

Findings will be reported to the participating ministries of health, the commissioners of this study, and to development finance partners, where applicable. Results will also be presented at local, national, and international conferences and disseminated via peer-review publications.

For peer review only - http://bmjopen.bmj.com/site/about/guidelines.xhtml

We aim to produce individual country case study manuscripts followed by a multiple case study synthesizing findings from the three study sites.

IV. Study significance

Research on the use of performance information in PHC systems is scarce; multi-country case studies in LMICs are inexistent to the best of our knowledge. The study presented here can contribute to an understanding of the contextual factors and organisational environments that enable or hinder the use of performance information in the PHC systems of El Salvador, Lebanon, and Malawi. Such knowledge can inform future research and contribute to improve the strategies used in LMIC settings to measure and manage PHC system performance.

Author contributions

The study was conceptualised by Wolfgang Munar, Martha Makwero and Fadi El-Jardali. The first draft was written by Syed Shabab Wahid, Wen-Chien Yang, and Wolfgang Munar, with inputs from Luckson Dullie, Martha Makwero and Fadi El-Jardali. All authors have read and approved the final manuscript.

Acknowledgement

We would like to acknowledge the Primary Health Care Research Consortium and for their support, in particular Doctor D. Praveen and Manushi Sharma. This work was supported, in whole, by the Bill & Melinda Gates Foundation [INV-000970]. Under the grant conditions of the Foundation, a Creative Commons Attribution 4.0 Generic License has already been assigned to the Author Accepted Manuscript version that might arise from this submission.

Funding Statement

This study is funded by the Bill and Melinda Gates Foundation through the Primary Health Care

For peer review only - http://bmjopen.bmj.com/site/about/guidelines.xhtml

Research Consortium hosted at the George Institute for Global Health in India [INV-000970]

Declaration of Interests

The researchers declare that they have no conflicts of interest.

V. References

1. Peiris D, Sharma M, Praveen D, Bitton A, Bresick G, Coffman M, et al. Strengthening primary health care in the COVID-19 era: a review of best practices to inform health system responses in low- and middle-income countries. WHO South-East Asia Journal of Public Health. 2021;10(3):6-25.

2. Friedrich M. Declaration of Astana emphasizes primary care as key to world health. Jama. 2018;320(23):2412-.

3. WHO, UNICEF. A vision for primary health care in the 21st century: towards universal health coverage and the Sustainable Development Goals. Geneva, Switzerland: WHO and UNICEF; 2018.

4. WHO. The world health report 2008: primary health care (Now more than ever). Geneva: World Health Organization; 2008.

5. Murray CJ, Frenk J. A framework for assessing the performance of health systems. Bulletin of the world Health Organization. 2000;78:717-31.

6. Pollitt C. Performance management 40 years on: a review. Some key decisions and consequences. Public Money & Management. 2018;38(3):167-74.

7. OECD. Measuring up: improving health system performance in OECD countries. Paris: OECD Publishing; 2002.

8. Fernandes G, Sridhar D. World Bank and the global financing facility. BMJ. 2017;358.

9. Eichler R, Agarwal K, Askew I, Iriarte E, Morgan L, Watson J. Performance-based Incentives to Improve Health Status of Mothers and Newborns: What Does the Evidence Show? Journal of Health Population and Nutrition. 2013;31(4):36-47.

10. Fan V, Denizhan Duran R. Grant Performance and Payments at the Global Fund. Washington, DC: Center for Global Development; 2013. Contract No.: 31.

11. Mokdad AH, Palmisano EB, Zuniga-Brenes P, Rios-Zertuche D, Johanns CK, Schaefer A, et al. Supply-side interventions to improve health: Findings from the Salud Mesoamerica Initiative. Plos One. 2018;13(4).

12. Broadbent J, Laughlin R. Performance management systems: A conceptual model. Manage Account Res. 2009;20(4):283-95.

13. Ferreira A, Otley D. The design and use of performance management systems: An extended framework for analysis. Manage Account Res. 2009;20(4):263-82.

14. Talbot C. Theories of performance: Organizational and service improvement in the public domain. New York, NY: Oxford University Press; 2010.

For peer review only - http://bmjopen.bmj.com/site/about/guidelines.xhtml

15. Pollitt C. The logics of performance management. Evaluation-Us. 2013;19(4):346-63.

16. Shore C, Wright S, Amit V, Brown J, Bruun Jensen C, Maguire M, et al. Audit culture revisited: Rankings, ratings, and the reassembling of society. Current Anthropology. 2015;56(3):431-2.

17. Eisenhardt KM. Agency theory: An assessment and review. Academy of Management Review. 1989;14(1):57-74.

18. Newton-Lewis T, Munar W, Chanturidze T. Performance management in complex adaptive systems: a conceptual framework for health systems. BMJ Global Health. 2021;6(7):e005582.

19. Davis JH, Schoorman FD, Donaldson L. Toward a stewardship theory of management. Academy of Management Review. 1997;22(1):20-47.

20. Walker RM, Lee MJ, James O, Ho SMY. Analyzing the Complexity of Performance Information Use: Experiments with Stakeholders to Disaggregate Dimensions of Performance, Data Sources, and Data Types. Public Administration Review. 2018;78(6):852-63.

21. Walker RM, Boyne GA, Brewer GA, editors. Public management and performance: Research directions. Cambridge, UK: Cambridge University Press; 2012.

22. Franco-Santos M, Lucianetti L, Bourne M. Contemporary performance measurement systems: A review of their consequences and a framework for research. Manage Account Res. 2012;23(2):79-119.

23. Levesque J-F, Sutherland K. What role does performance information play in securing improvement in healthcare? A conceptual framework for levers of change. BMJ open. 2017;7(8):e014825.

24. Kanfer R, Frese M, Johnson RE. Motivation Related to Work: A Century of Progress. Journal of Applied Psychology. 2017;102(3):338-55.

25. Van Dooren W, Bouckaert G, Halligan J, editors. Performance measurement in the Public Sector. Second ed: Routledge; 2015.

26. Pandey SK. Performance Information Use: Making Progress But a Long Way to Go. Public Perform Manag. 2015;39(1):1-6.

27. Moynihan DP, Pandey SK. The Big Question for Performance Management: Why Do Managers Use Performance Information? Journal of Public Administration Research and Theory. 2010;20(4):849-66.

28. EPOC. Effective Practice and Organisation of Care (EPOC). EPOC taxonomy Cochrane Collaboration. Norwegian Satellite2015 [cited 2019 October 17, 2019]. Available from: https://epoc.cochrane.org/epoc-taxonomy.

29. Munar W, Snilstveit B, Aranda LE, Biswas N, Baffour T, Stevenson J. Evidence gap map of performance measurement and management in primary healthcare systems in low-income and middle-income countries. BMJ Global Health. 2019;4(Suppl 8):e001451.

30. Franco-Santos M, Otley D. Reviewing and Theorizing the Unintended Consequences of Performance Management Systems. International Journal of Management Reviews. 2018;20(3):696-730.

31. Moynihan DP. The dynamics of performance management: Constructing information and reform. 12 ed. Washington, DC: Georgetown University Press; 2008 March 11, 2008. 224 p.

For peer review only - http://bmjopen.bmj.com/site/about/guidelines.xhtml

32. Vahamaki J, Verger C. Learning from Results-Based Management evaluations and reviews. Paris: OECD; 2019.

33. Leon N, Balakrishna Y, Hohlfeld A, Odendaal WA, Schmidt B-M, Zweigenthal V, et al. Routine Health Information System (RHIS) improvements for strengthened health system management. Cochrane Database of Systematic Reviews. 2020(8).

34. Aqil A, Lippeveld T, Hozumi D. PRISM framework: a paradigm shift for designing, strengthening and evaluating routine health information systems. Health Policy and Planning. 2009;24(3):217-28.

35. Hotchkiss DR, Aqil A, Lippeveld T, Mukooyo E. Evaluation of the Performance of Routine Information System Management (PRISM) framework: evidence from Uganda. BMC Health Services Research. 2010;10.

36. Wagenaar BH, Hirschhorn LR, Henley C, Gremu A, Sindano N, Chilengi R, et al. Data-driven quality improvement in low-and middle-income country health systems: lessons from seven years of implementation experience across Mozambique, Rwanda, and Zambia. Bmc Health Services Research. 2017;17.

37. Hoxha K, Hung YW, Irwin BR, Grépin KA. Understanding the challenges associated with the use of data from routine health information systems in low-and middle-income countries: A systematic review. Health Information Management Journal. 2020:1-14.

38. Contandriopoulos D, Champagne F, Denis J-L. The multiple causal pathways between performance measures' use and effects. Med Care Res Rev. 2014;71(1):3-20.

39. Contandriopoulos D, Lemire M, Denis JL, Tremblay E. Knowledge Exchange Processes in Organizations and Policy Arenas: A Narrative Systematic Review of the Literature. Milbank Quarterly. 2010;88(4):444-83.

40. Damschroder LJ, Aron DC, Keith RE, Kirsh SR, Alexander JA, Lowery JC. Fostering implementation of health services research findings into practice: a consolidated framework for advancing implementation science. Implementation Science. 2009;4(1):1-15.

41. Heinrich CJ, Marschke G. Incentives and Their Dynamics in Public Sector Performance Management Systems. J Policy Anal Manag. 2010;29(1):183-208.

42. Deci EL, Olafsen AH, Ryan RM. Self-Determination Theory in Work Organizations: The State of a Science. Annual Review of Organizational Psychology and Organizational Behavior, Vol 4. 2017;4:19-43.

43. Gagne M, Deci EL. Self-determination theory and work motivation. Journal of Organizational Behavior. 2005;26(4):331-62.

44. Cerasoli CP, Nicklin JM, Ford MT. Intrinsic Motivation and Extrinsic Incentives Jointly Predict Performance: A 40-Year Meta-Analysis. Psychological Bulletin. 2014;140(4):980-1008.

45. Colquhoun HL, Carroll K, Eva KW, Grimshaw JM, Ivers N, Michie S, et al. Advancing the literature on designing audit and feedback interventions: identifying theory-informed hypotheses. Implementation Science. 2017;12(1):117.

46. Weick KE, Sutcliffe KM, Obstfeld D. Organizing and the process of sensemaking. Organization Science. 2005;16(4):409-21.

47. Maitlis S, Christianson M. Sensemaking in organizations: Taking stock and moving forward. Academy of Management Annals. 2014;8(1):57-125.

For peer review only

For peer review only - http://bmjopen.bmj.com/site/about/guidelines.xhtml

48. Argyris C, Schon DA. Organizational learning: A theory of action perspective. Reis. 1997(77/78):345-8.

49. Argyris C. Double loop learning in organizations. Harvard Bus Rev. 1977;55(5):115-25.

50. Gilson L. Health policy and systems research: a methodology reader. Geneva: World Health Organization; 2012.

51. Yin R, K. Applications of case study research. Third ed. Thousand Oaks, USA: Sage Publications; 2011 June 21, 2011. 264 p.

52. Yin R, K. Case study research and applications: Design and methods. Thousand Oaks, CA.: Sage Publications; 2018.

53. World-Bank. The World Bank in El Salvador Washington, DC. USA2019 [Available from: https://www.worldbank.org/en/country/elsalvador/overview.

54. IADB. El Salvador Washington, DC. USA2019 [Available from: https://www.iadb.org/en/alianza-para-la-prosperidad/el-salvador.

55. Munar W, Wahid SS, Mookherji S, Innocenti C, Curry LA. Team-and individual-level motivation in complex primary care system change: A realist evaluation of the Salud Mesoamerica Initiative in El Salvador. Gates Open Research. 2018;2.

56. Bernal P, Martinez S. In-kind incentives and health worker performance: Experimental evidence from El Salvador. Journal of Health Economics. 2020;70:102267.

57. World Bank. Lebanon Country Overview Washington, DC2020 [Available from: https://www.worldbank.org/en/country/lebanon/overview

58. UNHCR. Lebanon. Time-series Syrian Refugees Registered by Cadastral in Lebanon (2012-2019) 2019 [Available from: https://data2.unhcr.org/en/documents/details/40569

59. Harake W, Abou Hamde NM. So When Gravity Beckons, the Poor Don't Fall. Washington, DC: The World Bank; 2019. Contract No.: 145454.

60. Ismaeel H, El Jamal N, Dumit N, Al-Chaer E. Saving the Suffering Lebanese Healthcare Sector: Immediate Relief while Planning Reforms. Paris: Arab Reform Initiative; 2020.

61. Hamadeh RS, Kdouh O, Hammoud R, Leresche E, Leaning J. Working short and working long: can primary healthcare be protected as a public good in Lebanon today? Conflict and health. 2021;15(1):1-9.

62. Hemadeh R, Kdouh O, Hammoud R, Jaber T, Khalek LA. The primary healthcare network in Lebanon: a national facility assessment. Eastern Mediterranean Health Journal. 2020;26(6-2020):700.

63. El- Jardali F, Fadlallah R, Matar L. Primary health care systems (PRIMASYS): comprehensive case study from Lebanon. Geneva: World Health Organization; 2017.

64. Ministry of Public Health L. Health Response Strategy: Maintaining Health Security, Preserving Population Health & Saving Children and Women Lives A New Approach 2016 & Beyond Beirut, Lebanon2016 [Available from: https://www.moph.gov.lb/userfiles/files/Strategic%20Plans/HealthResponseStrategy-updated.pdf

65. Ministry of Public Health L. Health Strategic Plan: Strategic Plan for the medium term (2016 to 2020) Beirut, Lebanon: Ministry of Public Health, Lebanon; 2016 [Available from: https://www.moph.gov.lb/en/Pages/9/11666/.

For peer review only - http://bmjopen.bmj.com/site/about/guidelines.xhtml

66. Ministry of Public Health L. MOPH Strategy 2025 Beirut, Lebanon2018 [cited 2021. Available from: https://www.moph.gov.lb/en/view/19754/ministry-of-public-health-strategy-health-2025-

67. Ministry of Public Health L. Health Reform in Lebanon Beirut, Lebanon2021 [Available from: https://www.moph.gov.lb/en/view/1279/health-reform-in-lebanon

68. World Bank. Malawi. Overview Washington, DC2021 [cited 2021. Available from: https://www.worldbank.org/en/country/malawi/overview#1.

69. WHO. Malawi Geneva2021 [cited 2021. Available from: https://www.afro.who.int/countries/malawi.

70. Makwero MT. Delivery of primary health care in Malawi. African Journal of Primary Health Care and Family Medicine. 2018;10(1):1-3.

71. Angwenyi V, Aantjes C, Kondowe K, Mutchiyeni JZ, Kajumi M, Criel B, et al. Moving to a strong (er) community health system: analysing the role of community health volunteers in the new national community health strategy in Malawi. BMJ global health. 2018;3(Suppl 3):e000996.

72. Patton MQ. Qualitative research and evaluation methods. Fourth ed. Thousand Oaks, CA: Sage publications; 2014. 806 p.

73. Malterud K, Siersma VD, Guassora AD. Sample size in qualitative interview studies: guided by information power. Qual Health Res. 2015;26(13):1753-60.

74. Hsieh H-F, Shannon SE. Three approaches to qualitative content analysis. Qual Health Res. 2005;15(9):1277-88.

75. O'Brien BC, Harris IB, Beckman TJ, Reed DA, Cook DA. Standards for reporting qualitative research: a synthesis of recommendations. Acad Med. 2014;89(9):1245-51.

76. Zhang Y, Zhang J, Li JJ. The effect of intrinsic and extrinsic goals on work performance: Prospective and empirical studies on goal content theory. Personnel Review. 2018;47(4):900-12.

77. Denzin NK, Lincoln YS. The Sage handbook of qualitative research. 5th ed. Thousand Oaks, CA: Sage Publications; 2011.

For peer review only

For peer review only - http://bmjopen.bmj.com/site/about/guidelines.xhtml

Figure Legend

Figure 1. Performance measurement and management system

Figure 2 - Theoretical framework

For peer review only

For peer review only - http://bmjopen.bmj.com/site/about/guidelines.xhtml

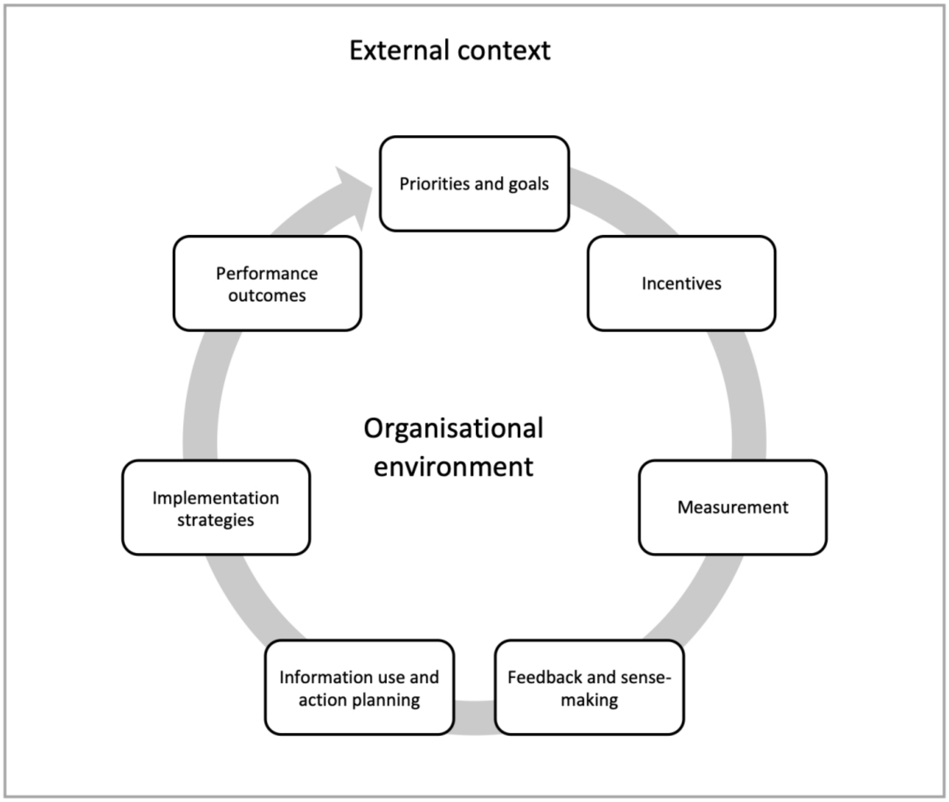

Figure 1. Performance measurement and management system

530x423mm (72 x 72 DPI)

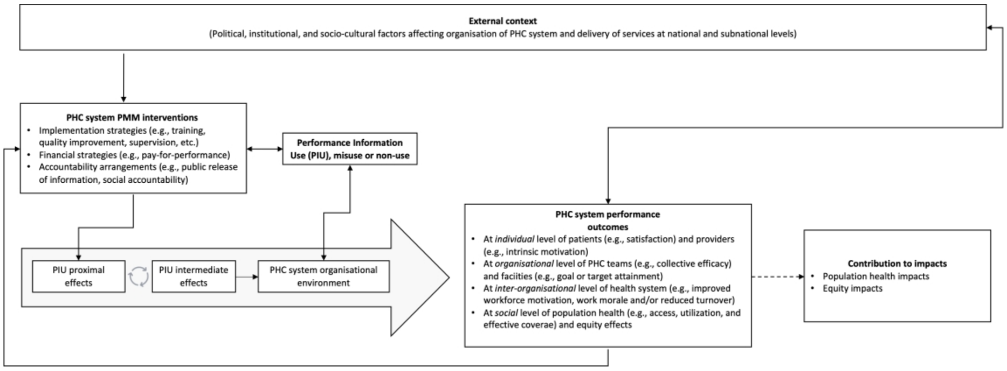

Figure 2. Theoretical framework

1615x584mm (72 x 72 DPI)

Supplementary file – Interview guidelines

For peer review only

For peer review only - http://bmjopen.bmj.com/site/about/guidelines.xhtml

El Salvador interview guidelines- PHC team members

Introduction.

For the past decade, the Salud Mesoamerica Initiative has been working in collaboration with the Ministry of Health to promote maternal and child health in El Salvador. The overall objective of this study is to assess the ways in which MCH performance information is collected and used at the local and national levels in El Salvador. The team obtained non-objection from the Ministry of Health to conduct this research. The findings will be used to develop better health care practices in El Salvador and hopefully contribute to better health outcomes for Salvadorans.

1. Do you recall participating (being interviewed) in 2018 for a study by (anonymized) and the Salud Mesoamerica Initiative?
2. Could you describe to me what you remember about the study (studies) in which you participated?

Section 1 Priorities and goals

1.1 How long have you been in this position?

1.2. What was your previous position?

1.3. Could you describe to me -in broad outline- the main activities you carry out in your current position?

1.4 What do you consider to be the priorities of the Ministry of Health in the primary health care system? What do you think of these priorities?

1.5. What are the priorities of your PHC team at this moment? How do they prioritize activities in the Unit to which you belong?

1.6. What is the role of the PHC teams in the health system of El Salvador?

1.7. Are you familiar with the Mesoamerican Health Initiative Program? If yes, is SMI currently supporting this Unit? Give me an example of how this support occurs? Based on your criteria, what has been the greatest contribution from SMI to this PHC team?

Section 2 - Performance Measurement

2.1. What is your perception of the work your PHC team is currently doing?

2.2. How do you know if your team is doing its job well or not?

2.3 The Salud Mesoamerica Initiative used to measure PHC team performance with some frequency. Rewards and recognition were often given to teams that were positively evaluated. Did you experience these?

Please describe those experiences.

How did you feel about those experiences (performance measurement and recognition)?

For peer review only - http://bmjopen.bmj.com/site/about/guidelines.xhtml

2.4. Are there other performance appraisal experiences?

2.5. How is your team's performance evaluated now?

Let's analyze the most recent example.

2.6. When did the appraisal occur?

2.7. Who did the evaluation?

2.8. How often is this done?

2.9. What data sources were used the last time one of these evaluations was conducted?

2.10. In the most recent evaluation of the team's performance, did the team receive any feedback?

2.11. How did you feel about this feedback?

2.12. How do you think the team felt? Elaborate on responses.

If feedback was provided:

2.13. How was the feedback provided? (Oral, written)

2.14. How often is feedback provided to your team about their performance?

2.15. How did you feel about this feedback?

2.16. What was done with the information contained in the feedback?

2.17. Were other team members made aware of the feedback? If yes, how was it shared?

2.18. Did the team do anything to correct their collective performance? Could you give me an example of an activity that was done?

If NO, comments or feedback were provided:

2.19. Would you like feedback on your team's performance?

Why?

2.20. Do you have any meetings with the MOH that you attend to discuss your team's performance?

If YES:

2.21. What is your opinion of these meetings, and why?

2.22. Are comparisons made between your team's performance and the performance of other teams?

Yes/no, please inquire:

2.23. How important would it be for you to attend these meetings? Do you feel it would be important to attend as a team? Why?

Section 3 - First Level of Care team performance management and individual work motivation

3.1. What aspects of your work as part of a PHC team are most rewarding to you?

For peer review only - http://bmjopen.bmj.com/site/about/guidelines.xhtml

3.1.1. Tell me about a recent work event that made you feel deeply rewarded or gratified.

3.1.2. Why did you feel this way? (Explore the 'why' in terms of emotions and feelings)

3.2. Sometimes the work is hard. Apart from COVID, what aspects of your work in a PHC team have been negative?

3.2.1. Tell me about a recent work event that affected you negatively.

3.2.2. Why did you feel this way? (Explore the 'why' in terms of emotions and feelings)

For peer review only

El Salvador decisionmakers at national and subnational levels

Introduction.

For the past decade, the Salud Mesoamerica Initiative has been working in collaboration with the Ministry of Health to promote maternal and child health in El Salvador. The overall objective of this study is to assess the ways in which MCH performance information is collected and used at the local and national levels in El Salvador. The team obtained non-objection from the Ministry of Health to conduct this research. The findings will be used to develop better health care practices in El Salvador and hopefully contribute to better health outcomes for Salvadorans.

Section 1 - Current Ministry of Health Primary Health Care Priorities

1.1. Tell me about the Ministry's priorities in the area of primary health care.

1.2. What are the strategic principles that guide the Ministry's PHC priorities?

Probe for:

1.2.1. Urban vs. rural; Comprehensive versus selective.

1.2.3. Efficiency and equity in the allocation.

1.2.4. Universalization versus targeting.

1.3. What role do the PHC teams now have in the functioning of the PHC system?

1.4. How well do you think PHC teams are doing their work?

1.5. What is the current role of SMI in relation to the priorities of the Ministry of Health in PHC?

Probe:

1.5.1. What is your perception of the value of MCH assistance to the MOH?

1.5.2. Could you give me an example?

Section 2 - Measuring PHC Team Performance

2.1 How does the Ministry evaluate the performance of the country's PHC system?

2.2 And in particular, how does the Ministry monitor the activities of the PHC teams?

2.3. Does the Ministry discuss any specific service delivery objectives with the teams?

2.3.1. What role do the teams play in the establishment of these objectives?

2.3.2. Describe the process (Investigate frequency, types of objectives, e.g., scope and/or facilities).

For peer review only - http://bmjopen.bmj.com/site/about/guidelines.xhtml

2.3.3. What performance indicators are now used?

Probe for:

- number of indicators used and request a list of key performance indicators in use.

2.3.4. Are performance targets used in all teams in the country? (If only the 75 teams in SMI's area of influence, ask why.)

2.4. In terms of service delivery, how does the Ministry evaluate the performance of the PHC teams?

Probe:

2.4.1. Using a recent example from a specific rural region, describe the process in detail.

2.4.2. What data sources did you use?

2.4.3. Who collects performance data from PHC teams?

2.4.4. Who analyzes the PHC team performance data?

2.4.5. How are the results presented to the PHC teams?

Section 3- Performance Management of PHC Teams

3.1. What are the PHC teams supposed to do with the results of the performance evaluation?

3.1.1. Does the ministry support the teams in understanding the results? How?

3.1.2. The Ministry has a long tradition of convening team managers and regional officers in frequent weekly or biweekly meetings to monitor progress. Is it still done? What are the results?

Section 4: Managing the overall performance of the PHC system

4.1 Beyond performance management of PHC teams, what else is the ministry doing with the results?

Probe:

4.1.1. Are the results used for planning? (If yes, ask for a recent example) 4.1.2.

4.1.2. Are the results used for budgeting? (If yes, ask for a recent example) 4.1.3.

4.1.3. Are the results used for any internal Ministry of Health reporting? (If yes, please ask for a recent example) 4.1.4.

4.1.4. Are the results used for MCH reporting? (If yes, please ask for a recent example) 4.2.

4.2. Does the MOH use aggregated data from PHC teams to adjust its priorities? How? Could you give me examples?

For peer review only - http://bmjopen.bmj.com/site/about/guidelines.xhtml

Lebanon interview guidelines - Health service providers and data collectors in the First Level of Care

Section 1- PHC Performance Measures

How is the aggregate performance of PHC system determined? Is there a performance assessment? What does it include?

Can you reflect on the processes and tools through which PHC system performance information is collected and processed? How would you characterize these?

Are there established performance indicators for PHC? If yes, can you share a sample of the performance indicators template (without any patient information or any other personally identifiable data)

How are the performance indicators selected? Which entity/department/unit/platform/team has input in this process (please provide only titles, not individual names)? Are these selected according to explicit criteria including usefulness, scientific soundness, reliability, representativeness, feasibility, accessibility?

Is there a guide/manual on how to calculate and interpret performance indicators? Does reporting on the performance indicators occurs on a regular basis?

How is information about performance measures communicated to you? For example, how did you first hear about them?

When a decision is made at the national level, how is it translated /monitored/implemented at subnational levels? Which entity/department/unit/platform/team is responsible for the communication from national to subnational level (please provide only titles, not individual names)? From subnational back to national level? How quickly does this happen?

What interventions or programs, if any, are implemented to ensure an enabling system environment for quality at level of PHC?

Registration and licensing; External evaluation/accreditation; Clinical governance; Public reporting and comparative benchmarking

For peer review only

Section 2- Performance Targets and Goals

2.1. How are performance targets for PHC set? Which entity/department/unit/platform/team is involved in the process (please provide only titles/department/unit, not individual names)? Are these typically set at a national, sub-national, or sub-regional level or left to the discretion of the facility?

Section 3- Data collection, analysis and sharing

3.1. Data collection

3.1.1. Is the data needed to construct the performance measure feasible to collect?

3.1.2. In what format is data collected? Is there a designated person to enter data/compile reports from the different units in the health facility?

3.1.3. What is the frequency of data collection? Is there a set data submission schedule?

3.1.4. Is there coordination of data collection across all PHC, e.g. via a unique identifier or a centralized data storage platform?

3.2. Data storage

3.2.1. In what database or file do you enter or store the data after you receive them? What computer program do you use? How frequently do you enter/store the data?

3.2.2. Any challenges in entering/storing the data? Any privacy issues when storing data?

3.3. Data aggregation

3.3.1 Do you perform any data aggregations? Which ones? Using which calculations? How often? Are there any challenges in aggregating data?

3.4. Data quality

3.4.1. What quality checks do you perform on the data? Is there a designated person to review the quality of compiled data prior to submission to the next level, e.g., to districts, to regional offices, to the central HMIS, etc.?

3.4.2. Are there any known issues with data quality?

3.5. Data reporting & sharing

3.5.1. Are there written guidelines available on reporting protocols, including the following: what they are supposed to report on; how reports are to be submitted, e.g., in what specific format; to whom the reports should be submitted and when the reports are due?

3.5.2. In what format do you share the data (type of form, file, database, aggregation levels)? Are data visuals prepared (graphs, tables, maps, etc., balanced scorecards) showing achievements toward targets (indicators, geographic and/or temporal trends, and situation data)?

3.5.3. With whom do you share the data? How often do you share the data?

For peer review only

3.5.4. How do you share the data (e.g., internet, email, USB flash drive, disk, hard copy)?

3.5.5. Are there any delays or challenges in preparing data & sending them?

Section 4- Performance Monitoring & Feedback

How is progress towards the performance targets monitored/tracked? Can you give us an example? Is this considered routine?

Is there a performance monitoring or management team?

Which entity/department/unit/platform/team is the most responsible for managing or tracking progress for performance measures? (please provide only titles, not individual names)

Which entity/department/unit/platform/team do they report to/who manages this process? (Please provide only titles, not individual names)

Which entity/department/unit/platform/team is in charge of the data pulls and reports? (please provide only titles, not individual names)

Which entity/department/unit/platform/team do the data analysts and the performance manager report to? (Please provide only titles, not individual names)

Section 5- HIS and data quality assessment

5.1. What specific information flows has the organization in place to support the performance management systems? Are systems interoperable so that information can be shared across geographic areas, types of facilities, etc.?

5.2. Are staff trained in how to use the information systems (not only collecting data, but also collating, analysing and interpreting data)

5.2.1. Do these staff typically have dedicated time allocated for using information systems and interpreting data?

5.2.2. Do they have capacity to explain the implications of the results of data analysis?

5.2.3. Are the information systems accessible to these individuals?

5.3. Are there data quality assessment mechanisms in place?

5.3.1. Are there written instructions/guidelines on how to perform a data quality review or data quality check?

5.3.2. Do data management staff conduct regular checks of the accuracy and completeness of data prior to submitting reports to the next level (using automated electronic checks, where appropriate)?

5.3.3. Does the health facility receive periodic feedback from higher levels (e.g., MOPH) on data quality?

For peer review only - http://bmjopen.bmj.com/site/about/guidelines.xhtml

5.3.4. What is your perception of the quality of existing data on PHC performance? How can it be improved?

Section 6- Supervision & Feedback on performance

Do you receive technical support or supervision in your work?

Which entity/department/unit/platform/team conducts supervision? What sort of support of qualifications do these individuals have? (please provide only titles, not individual names)

Which entity/department/unit/platform/team receives supervision? Which ones do not? (please provide only titles, not individual names)

Where do supervisory visits take place and how long do supervisory visits last?

What sort of activities do supervisory visits cover? Does the supervisor use a standard checklist for assessment?

How frequently does supervision occur?

Is there an established schedule for supervision, or do visits need to be requested?

Does the supervisor send report/ written feedback on the past supervisory visit(s)?

Do you get feedback on your performance? How? Frequency? What does it look like? How would you improve this process?

Feedback on data quality (including data accuracy, reporting timeliness, and/or report completeness)

Feedback on service performance based on reported data (e.g., appreciation/acknowledgement of good performance; resource allocation/mobilization)

What, if any, feedback loops exist between the facility level, regional, sub-national level and national level and how do they function?

What rewards — financial and/or non-financial — will managers and other employees gain by achieving performance targets or other assessed aspects of performance (and what penalties will they suffer by failing to achieve them)?'

Section 7- Implementation of quality improvement activities

Are discussions often held to review key performance targets (tracking progress against targets) based on performance measure information?

Are quality improvement activities implemented to take corrective action?

How are the objectives of quality improvement activities set?

For peer review only

For peer review only - http://bmjopen.bmj.com/site/about/guidelines.xhtml

Which entity/department/unit/platform/team initiates these activities? (please provide only titles, not individual names)

Which entity/department/unit/platform/team monitors them? (please provide only titles, not individual names)

Section 8- Overall perceptions on collection of performance measure information

8.1. To what extent to do you agree with the following:

I can use data for identifying performance gaps and setting targets

I feel discouraged when the data that I collect/record are not used for taking action (either for monitoring or decision making)

I find collecting/recording data to be tedious (i.e., repetitive or duplicative)

I find that the data that I collect burdens my workload, making it difficult for me to complete my other duties

Collecting data is meaningful/useful for me

I feel that the data I collect are important for monitoring the performance of the health services provided at my facility/unit

My work of collecting data is appreciated and valued by decision-makers at national and subnational level

For peer review only

Lebanon decisionmakers at national and subnational levels

Section 1- PHC Performance Measures

How is the aggregate performance of PHC systems measured (organizational and whole-system level)?

How do you track progress toward goals? Can you show us an example?

Which entity/department/unit/platform/team are most responsible for managing or tracking progress for performance measures? (please provide only titles, not individual names)

Which entity/department/unit/platform/team do they report to/who manages this process? (please provide only titles, not individual names)

Which entity/department/unit/platform/team is in charge of the data pulls and reports? (please provide only titles, not individual names)

Which entity/department/unit/platform/team do the data analysts and the performance manager report to? (please provide only titles, not individual names)

What rewards — financial and/or non-financial — will managers and other employees gain by achieving performance targets or other assessed aspects of performance (and what penalties will they suffer by failing to achieve them)?

Section 2- Awareness of data flow at different levels

2.1. Are you aware of how PHC performance measurement information is collected and processed at different levels?

2.2. Can you reflect on data flow from facility level to district/subnational to MoPH? What is your perception of the process?

Section 3- Data format

3.1. In what format do you receive performance measure information?

3.1.1. Are data visuals (e.g. summary tables, graphs, geographic information system, maps, pivot tables, decision support systems, etc.) showing achievements toward targets (indicators, geographic and/or temporal trends, and situation data) prepared and used to display information at MOPH level and within health facilities. They are up to date and clearly understood

3.1.2. Are reports or bulletins (annual, quarterly, etc.) periodically produced based on an analysis of performance measurement information and distributed to key stakeholders? Do they contain discussions and decisions/recommendations based on key performance targets?

For peer review only - http://bmjopen.bmj.com/site/about/guidelines.xhtml

3.1.3. Are there challenges with interpreting the information?

3.2.3. What is the frequency of receiving performance measure information? Is there a set schedule for receiving such information? What is your perception of the process?

3.2. How do you receive the performance measure information (e.g., internet, email, USB flash drive, disk, hard copy)? In what database or file is data stored or entered after you receive them?

3.3. What quality checks do you perform on the data? Are there any known issues with data quality?

3.4. How could (or should) health information products look like for policy-makers? and at what level of disaggregation and/or in which quality?

3.5. With whom do you share the data? In what format do you share the data (type of form, file, database, aggregation levels)? How do you share the data (e.g., internet, email, USB flash drive, disk, hard copy)?

Section 4- Feedback on data received

4.1. When a decision is made at the national level, how is it translated /monitored/implemented at subnational levels? Which entity/department/unit/platform/team is responsible for the communication from national to subnational level (please provide only titles, not individual names)? From subnational back to national level? How quickly does this happen?

4.2. Is feedback systematically provided to all sub-reporting units on the quality of their reporting (that is, accuracy, completeness, and timeliness)?

4.3. Is feedback systematically provided to all sub-reporting units on the use of performance measure information for decision-making?

Section 5- Decision-making process

5.1. How are decisions made at your institution? What factors have strong influence on decision-making process:

Personal preference of decision makers

Superiors' directives

Evidence/facts/data (RHIS data)

Funding directives from higher levels

Political considerations

Official health sector strategic objectives

Locally identified health needs of the population

The relative cost of interventions

For peer review only - http://bmjopen.bmj.com/site/about/guidelines.xhtml

5.2. Is there a mandate to use data to inform decision-making? What incentive structures are in place to promote the use of data in decision-making? Are there any consequences for NOT using data to inform critical decision-making?

Section 6- Use of data in decision-making process

6.1. Can you reflect on the processes through which decision makers (at national and district levels) make sense of, and use PHC system performance information? What is your perception of the process?

6.2. Do the existing performance measure information respond to policymakers' priorities and needs? Are data producers and users brought together periodically to discuss ways of making routine data more relevant to policy makers and planners and to enhance the understanding of routine health statistical findings?

6.3. How is aggregate PHC system performance information used by decision makers at the national and district level?

6.4. Were any decisions made based on the discussions of the performance measurement information? Such as:

Advocacy for policy, programmatic, or strategic decisions

Formulation of plans

Budget preparation/reallocation

Medicine supply and drug management

Human resource management (training, reallocation, etc.)

Promotion of service quality/improvement, equity

Section 7- Impact

7.1. Would your organizational priorities be different without performance measures? How? How do measures affect your relationship or interactions with staff?

7.2. How have performance measures changed the way you operate?

7.3. How do performance measures affect policy decisions? Budget decisions? Staff decisions? Scheduling? Education/training/orientation?

7.4. To what extent is (staff) compensation linked to performance measures? Can you explain how that works? How does this affect your perception of performance measures?

For peer review only - http://bmjopen.bmj.com/site/about/guidelines.xhtml

Section 8- Perceptions on data use culture

8.1. What is your perception of the prevailing culture for data use at your institution?

Probes:

A culture of information use is promoted by policy leaders and decision-makers, and is reflected in the use of facility and community-based data in planning, monitoring, and evaluation reports

Performance measure information is readily available in written periodic reports or bulletins that pulls together and analyses critical health information from all subsystems

Senior managers and policymakers demand complete, timely, accurate, relevant and validated HIS information

Policy and decision makers regularly use performance measure information to evaluate performance and set policies on health.

Data visualizations are widely used to display information at subnational health administrative offices and health facilities. They are up to date and clearly understood

Performance measurement information is demonstrably used in the national planning and in the resource-allocation processes (e.g., for annual integrated development plans, medium-term expenditure frameworks, long-term strategic plans, and annual health sector reviews)

Performance measure information is widely used, by sub-national management teams to set resource allocation in the annual budget processes

Performance measure information is used to advocate for equity and increased resources to disadvantaged groups and communities

Managers at all levels use health information for health service delivery management, continuous monitoring, and periodic evaluation

Section 9- Big Picture

9.1. How would you characterize the use of performance health information in Lebanon? All in all, do you think there is too much/not enough emphasis on performance measures?

9.2. What are the challenges that hinder the use of performance measurement information to inform health policymaking and decision-making processes?

9.3. Do you have suggestions on how to improve the role of performance measure information in informing the policymaking process?

Relevance

Quality

Timeliness/Accessibility

Analysis and interpretation

For peer review only - http://bmjopen.bmj.com/site/about/guidelines.xhtml

Format

9.4. Can you suggest up to three policies/ strategies/mechanisms that can help improve your institution's capacity to use data in policy and program development and implementation?

For peer review only

For peer review only - http://bmjopen.bmj.com/site/about/guidelines.xhtml

Malawi – Interview guide for Decision Makers

Participant details

Facilitator's Name: ______________________Date of the interview ______________

Name of the Facility:___________________ Location ______________

Respondent's age____________________ Respondent's Sex_________

Respondent's Position _________________ Qualification______________

Respondent's years of experience_____________________________

Introduction

What is your role in this organization?

What is your qualification

How long have you worked in this organization?

How long have you worked at your current position?

What are the main priorities of MOH in PHC?

What are your PHC priorities?

Currently, how can you rate your impact in PHC? Is your performance improving or not? How do you know?

Knowledge about data collection and use

Do you know how PHC data is collected at your institution?

If yes, may you briefly explain how it is done?

Do you think PHC data is collected in honest and effective manner?

Why is data collection and use important in your organization?

How is data utilized in policy formulation?

Performance Measurement

Please describe the ways in which the performance of PHC at your level is evaluated? Specifically, let us look at

Who does the evaluation?

When did the evaluation happen?

For peer review only - http://bmjopen.bmj.com/site/about/guidelines.xhtml

For peer review only

What data was used to assess the team's performance

How frequently is this done

When the performance assessment is complete, what type of feedback do you receive (oral or written)

How frequently do you receive the feedback about your performance?

What do you do with the feedback you receive about the PHC performance? May you give an example of a recent situation in which you used the performance results in your daily practice and services

Probe on: a. planning b. budgeting c. policy formulation

Do you have any specific example of a situation in which PHC performance results did not help you?

What do you think are the challenges associated with performance measurement?

What do you think can be done to address the challenges?

Factors affecting data driven decision making (DDDM)

May you briefly explain the things you consider when making a decision in your organization?

Probe on: data consultation, political factors, group/personal influence etc

Why are these things important in decision making?

Have you ever made important decision without relying on the available data?

If yes, why was data not relevant for that decision?

If not, why is relying on data important?

Are there factors/reasons for making a decision without relying on data?

Probe on: lack of data authenticity/trustworthiness, political influence, lack of understanding of data, lack of time to study the data, negligence etc

What do you think are the challenges associated with data driven decision making?

What do you think can be done to address the challenges?

Theme 3: Policies on data driven decision making

Are there policies that guide managers on data driven decision making?

If not, what drives managers to make a decision by relying on data?

Are all policies in PHC made in consultation with data?

If not, why?

What could be the result of formulating a policy without consulting data?

What does the ministry do?

Challenges and recommendations

Do you have any challenges on data collection and use in your organizations?

For peer review only - http://bmjopen.bmj.com/site/about/guidelines.xhtml

If yes, what are the challenges?

Probe on: lack of understanding of the data, bulkiness of the data, lack of time to study the data, lack of data authenticity, political interference etc

What do you think should be done to improve data collection and use in your organization?

Probe on: a. what should the data collectors and users do?

b. what should the government do?

Thank you very much. This is the end of our discussions.

For peer review only

For peer review only - http://bmjopen.bmj.com/site/about/guidelines.xhtml

Reporting checklist for qualitative study.

Based on the SRQR guidelines.

Instructions to authors

Complete this checklist by entering the page numbers from your manuscript where readers will find each of the items listed below.

Your article may not currently address all the items on the checklist. Please modify your text to include the missing information. If you are certain that an item does not apply, please write "n/a" and provide a short explanation.

Upload your completed checklist as an extra file when you submit to a journal.

In your methods section, say that you used the SRQR reporting guidelines, and cite them as:

O'Brien BC, Harris IB, Beckman TJ, Reed DA, Cook DA. Standards for reporting qualitative research: a synthesis of recommendations. Acad Med. 2014;89(9):1245-1251.

		Reporting Item	Page Number
	#1	Concise description of the nature and topic of the study identifying the study as qualitative or indicating the approach (e.g. ethnography, grounded theory) or data collection methods (e.g. interview, focus group) is recommended	10-11
	#2	Summary of the key elements of the study using the abstract format of the intended publication; typically includes background, purpose, methods, results and conclusions	2-3
Problem formulation	#3	Description and signifcance of the problem / phenomenon studied: review of relevant theory and empirical work; problem statement	4-10
Purpose or research question	#4	Purpose of the study and specific objectives or questions	7
Qualitative approach and research	#5	Qualitative approach (e.g. ethnography, grounded theory, case study, phenomenolgy, narrative research) and	7-11

For peer review only

paradigm		guiding theory if appropriate; identifying the research paradigm (e.g. postpositivist, constructivist / interpretivist) is also recommended; rationale. The rationale should briefly discuss the justification for choosing that theory, approach, method or technique rather than other options available; the assumptions and limitations implicit in those choices and how those choices influence study conclusions and transferability. As appropriate the rationale for several items might be discussed together.	
Researcher characteristics and reflexivity	#6	Researchers' characteristics that may influence the research, including personal attributes, qualifications / experience, relationship with participants, assumptions and / or presuppositions; potential or actual interaction between researchers' characteristics and the research questions, approach, methods, results and / or transferability	n/a
Context	#7	Setting / site and salient contextual factors; rationale	11-13
Sampling strategy	#8	How and why research participants, documents, or events were selected; criteria for deciding when no further sampling was necessary (e.g. sampling saturation); rationale	13-15
Ethical issues pertaining to human subjects	#9	Documentation of approval by an appropriate ethics review board and participant consent, or explanation for lack thereof; other confidentiality and data security issues	17
Data collection methods	#10	Types of data collected; details of data collection procedures including (as appropriate) start and stop dates of data collection and analysis, iterative process, triangulation of sources / methods, and modification of procedures in response to evolving study findings; rationale	13-16
Data collection instruments and technologies	#11	Description of instruments (e.g. interview guides, questionnaires) and devices (e.g. audio recorders) used for data collection; if / how the instruments(s) changed over the course of the study	14, Appendix
Units of study	#12	Number and relevant characteristics of participants, documents, or events included in the study; level of	14

For peer review only

		participation (could be reported in results)	
Data processing	#13	Methods for processing data prior to and during analysis, including transcription, data entry, data management and security, verification of data integrity, data coding, and anonymisation / deidentification of excerpts	15-16
Data analysis	#14	Process by which inferences, themes, etc. were identified and developed, including the researchers involved in data analysis; usually references a specific paradigm or approach; rationale	15-16
Techniques to enhance trustworthiness	#15	Techniques to enhance trustworthiness and credibility of data analysis (e.g. member checking, audit trail, triangulation); rationale	16
Syntheses and interpretation	#16	Main findings (e.g. interpretations, inferences, and themes); might include development of a theory or model, or integration with prior research or theory	n/a
Links to empirical data	#17	Evidence (e.g. quotes, field notes, text excerpts, photographs) to substantiate analytic findings	n/a
Intergration with prior work, implications, transferability and contribution(s) to the field	#18	Short summary of main findings; explanation of how findings and conclusions connect to, support, elaborate on, or challenge conclusions of earlier scholarship; discussion of scope of application / generalizability; identification of unique contributions(s) to scholarship in a discipline or field	n/a
Limitations	#19	Trustworthiness and limitations of findings	n/a
Conflicts of interest	#20	Potential sources of influence of perceived influence on study conduct and conclusions; how these were managed	n/a
Funding	#21	Sources of funding and other support; role of funders in data collection, interpretation and reporting	18

The SRQR checklist is distributed with permission of Wolters Kluwer © 2014 by the Association of American Medical Colleges. This checklist can be completed online using https://www.goodreports.org/, a tool made by the EQUATOR Network in collaboration with Penelope.ai

For peer review only
